# Protein kinase A activation alleviates cataract formation via increased gap junction intercellular communication

**DOI:** 10.1016/j.isci.2023.106114

**Published:** 2023-02-02

**Authors:** Yu Du, Yuxin Tong, Yumeng Quan, Guangyan Wang, Hongyun Cheng, Sumin Gu, Jean X. Jiang

**Affiliations:** 1 Department of Ophthalmology, Lanzhou University Second Hospital; Second Clinical School, Lanzhou University, Lanzhou, Gansu, 730000, China; 2 Department of Biochemistry and Structural Biology, University of Texas Health Science Center, San Antonio, TX 78229-3900, USA; 3 Department of Ophthalmology, First Affiliated Hospital of Xi’an Jiaotong University, Xi’an, Shaanxi, China

**Keywords:** Physiology, Molecular physiology, Molecular biology

## Abstract

Cataract is the leading cause of blindness worldwide. Here, we reported a potential, effective therapeutic mean for cataract prevention and treatment. Gap junction communication, an important mechanism in maintaining lens transparency, is increased by protein kinase A (PKA). We found that PKA activation reduced cataracts induced by oxidative stress, increased gap junctions/hemichannels in connexin (Cx) 50, Cx46 or Cx50 and Cx46 co-expressing cells, and decreased reactive oxygen species (ROS) levels. However, ROS reduction was shown in wild-type, Cx46 and Cx50 knockout, but not in Cx46/Cx50 double KO lens. In addition, PKA activation protects lens fiber cell death induced by oxidative stress via hemichannel-mediated glutathione transport. Connexin deletion increased lens opacity induced by oxidative stress associated with reduction of anti-oxidative stress gene expression. Together, our results suggest that PKA activation through increased connexin channels in lens fiber cell decreases ROS levels and cell death, leading to alleviated cataracts.

## Highlights

• Protein kinase A (PKA) activation reduces mouse cataracts induced by oxidative stress
• PKA increases gap junctions/hemichannels and decreases reactive oxygen species
• PKA activation protects lens fiber cell via hemichannel-mediated glutathione transport
• PKA activation via increased connexin channels in lens fiber cell alleviates cataracts

## Graphical abstract

**Figure:**
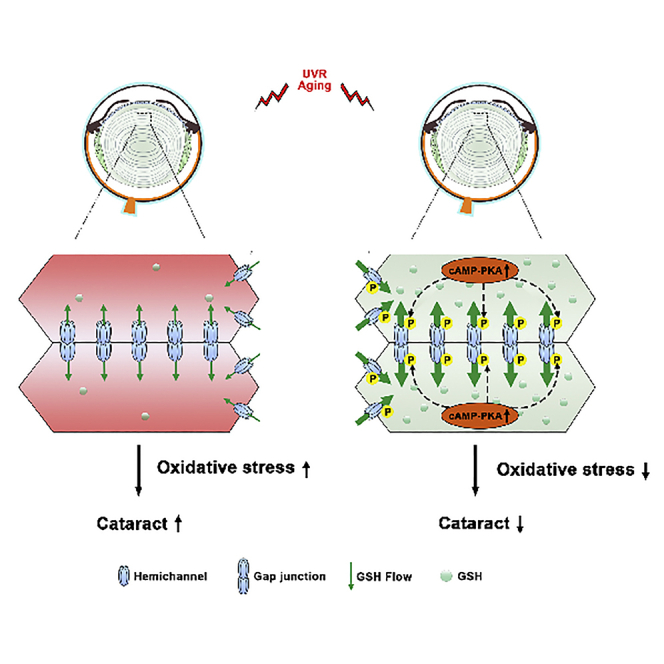

## Introduction

The lens is a transparent and avascular organ composed of capsule, epithelium, and cortical and nuclear fibers. The epithelial cells form a layer at the anterior lens surface under the capsule, where they continuously proliferate and differentiate at the equator region, differentiating into lens fiber cells. The organized fiber cells form the bulk of the lens, divided into nuclear fibers located in the center and the newly formed cortical fibers at the outer part of the lens.^1^ The unique structure and composition of the lens define its physiological function in transmitting and focusing light on the retina.^2^ Cataract, the naturally transparent lens, becoming cloudy or opacity, is a common eye disease and is also the leading cause of blindness and has a significant impact on global public health.^3^ At present, the standard care is surgical intervention; however, it is associated with higher economic cost^4^ and limitations, especially in areas with low resources or patients ill-equipped for cataract surgery.

The main cause of cataracts is oxidative stress. Throughout our life, lenses are continuously exposed to oxidative environments and prone to oxidative damage, such as exposure to UV radiation (UVR) and harmful chemicals, gradually leading to cataract formation.^5^^,^^6^ Another major source of reactive oxygen species (ROS) is hydrogen peroxide (H_2_O_2_), which has a 3-fold increase in the aqueous humor of patients with severe cataracts.^7^ Therefore, seeking a non-surgical method to prevent and control cataracts has become an unmet medical need. *Moringa oleifera* stem extract has been reported to alleviate oxidative stress-induced cataract formation.^8^ The exogenous antioxidant interventions could delay and/or prevent the progression of lens cataract,^9^ but the underlining mechanism remains obscure.

Connexins (Cxs) are crucial for lens transparency. Cxs formed gap junctions (GJs)^10^ and Cx hemichannels (HCs)^11^ are involved in the exchange of substances and metabolites between the lens cells or its microenvironment, respectively, and its extensive network is vital for maintaining osmotic and lens transparency.^11^^,^^12^^,^^13^ These channels are permeable to ions, small metabolites, and second messenger molecules and can be regulated by phosphorylation and redox changes.^14^ Three connexins are expressed in the mammalian lens; Cx43 and Cx50 in lens epithelium, and Cx46 and Cx50 are abundantly expressed in lens fibers. Cx50 and Cx46 form both homomeric and heteromeric Cx HCs.^15^^,^^16^^,^^17^^,^^18^ Cx mutations are a leading cause of congenital cataracts.^19^ In addition, Cx50 and Cx46 gene knockout (KO) lens develop nuclear cataracts,^20^ and Cx50 KO also exhibits small eyeballs and lenses, microphthalmia.^21^^,^^22^^,^^23^^,^^24^

Our previous studies have shown that Cx50 HCs and Cx46 HCs transport glutathione (GSH) and protect lens fiber cells against oxidative insults.^25^ In addition, we have shown that protein kinase A (PKA) activation enhanced both Cx50 GJs and HCs functions by stabilizing the channel in a more conductive configuration,^26^ indicating that PKA activation may enhance Cx GJs and HCs functions to support the delivery of antioxidants and protect the lens from oxidative stress.

In this study, we observed that PKA activators reduced intracellular ROS levels by enhancing Cx function, thereby decreasing lens fiber cell apoptosis. More importantly, we found that PKA activators alleviated cataracts in mouse lens induced by H_2_O_2_ and UVB, and this effect was impeded in Cx46/Cx50 dKO mouse lens, but not in Cx50 or Cx46 single KO due to the expression and compensatory roles of Cxs.

## Results

### PKA activation enhances HCs function and protects lens fiber cells against H_2_O_2_-induced cell death

**Figure 1:**
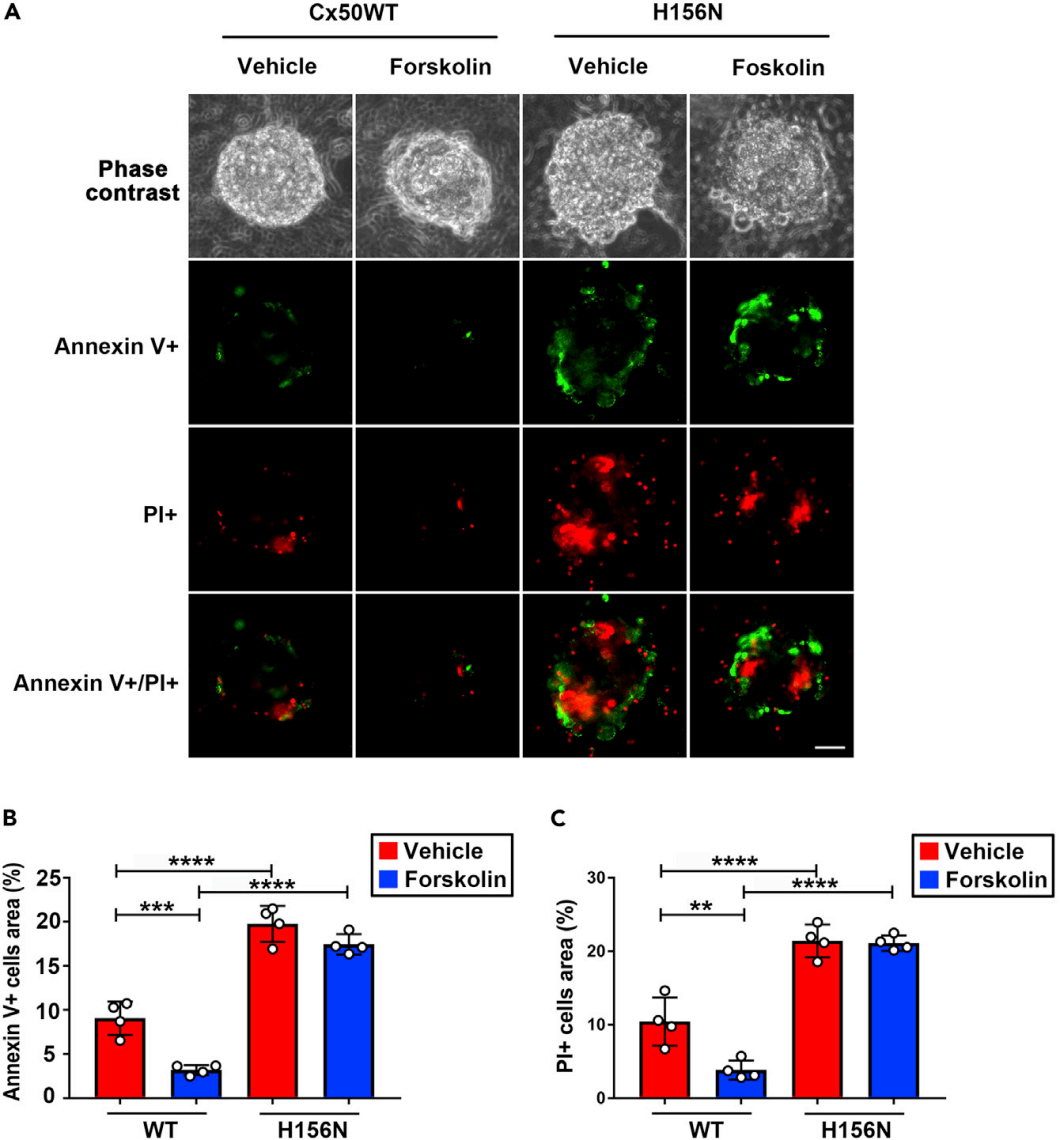
PKA activation protects lens fiber cells against H_2_O_2_-induced cell death via functional Cx50 HCs and GSH transport (A–C) Primary chick lens cell cultures were infected with high-titer recombinant RCAS(A) retroviruses containing Cx50, or Cx50 mutants H156N, and then treated with or without PKA activator: forskolin (1 μM) for 2 h before being subjected to fluid flow shear stress (FFSS) at 1 dyne/cm^2^ for 30 min, incubated with 1 mM GSH for 10 min and treated with 50 μM H_2_O_2_ for 4 h. Cell apoptosis and necrosis were detected using Dead Cell Apoptosis Kit. The percentage of cells under apoptosis (FITC-Annexin V+) (B) and PI + cells (C) was quantified (n = 4). Scale bar: 50 μm ∗∗p < 0.01, ∗∗∗p < 0.001, ∗∗∗∗p < 0.0001 (two-way ANOVA). At least three microphotographs of fluorescence fields were captured by a 20X microscope (Keyence BZ-X710) with a FITC filter and a rhodamine filter.

Our previous study showed that PKA activation enhanced both Cx50 GJs and HCs functions.^26^ In addition, uptake of GSH by Cx HCs decreases H_2_O_2_- and UVB-induced cell apoptosis and necrosis.^25^ To investigate whether PKA activation can activate HCs and alleviate cell apoptosis and necrosis, we used H_2_O_2_, an oxidative stress inducer (a central redox signaling molecule and a fast-acting oxidizing reagent^27^) to treat primary chick lens cells in the absence or presence of forskolin, a PKA activator. Cells under apoptosis and necrosis were detected using FITC–Annexin V Apoptosis Detection Kit (green for apoptotic (Annexin V+) and red for necrotic (PI+)) (Figure 1A). The percentage of apoptotic (Annexin V+) cells was significantly decreased in forskolin-treated groups compared to the vehicle-treated control (Ctrl) group, whereas inhibition of Cx HCs by a dominant-negative H156N mutant attenuated this effect (Figures 1B and 1C). These results suggest that forskolin promotes HCs function and protects lens fiber cells against H_2_O_2_-induced cell death.

### Cx deletion increased cataract formation induced by H_2_O_2_ and UVB

**Figure 2:**
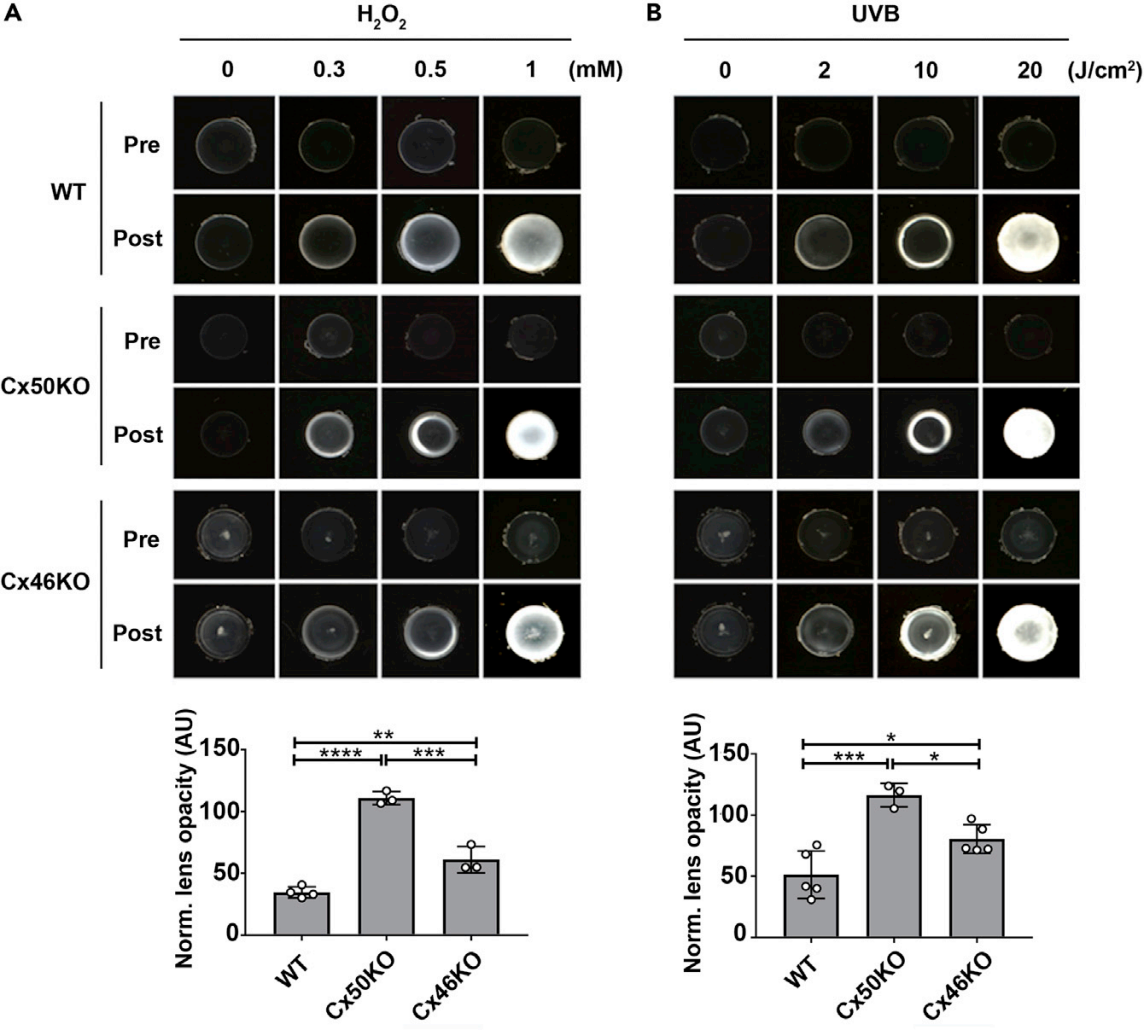
Deletion of connexin expression in the lens increased cataract formation induced by H_2_O_2_ and UVB (A) The lenses of WT, Cx50KO, and Cx46KO mice were dissected and kept transparent in culture media for 24 h at 37°C before being treated with different concentrations (0, 0.3, 0.5, and 1 mM) of H_2_O_2_. The lens opacity was measured with a treatment of 0.5 mM H_2_O_2_ (lower panel). Images were taken at identical magnification using a dissecting microscope. The data are presented as the mean ± SEM. (n ≥ 3). ∗∗, p < 0.01; ∗∗∗, p < 0.001; ∗∗∗∗, p < 0.0001 (One-way ANOVA). (B) WT, Cx50KO, and Cx46KO lens were treated with different intensities (0, 2, 10, and 20 J/cm^2^) of UVB radiation. The lens intensity was measured with 10 J/cm^2^ UVB radiation (lower panel). The data are presented as the mean ± SEM. (n ≥ 3). ∗, p < 0.05; ∗∗∗, p < 0.001 (One-way ANOVA).

We used two forms of major oxidative stress, H_2_O_2_ and UVR,^6^^,^^28^ to induce cataract formation in mouse lenses. In the control groups without H_2_O_2_ or UVB treatment, lenses maintained their original transparency for more than 1 week under our experimental condition. Different concentrations of H_2_O_2_ were used to induce cataracts in wild type (WT), Cx50KO, and Cx46KO mouse lenses. The increased opacity intensity induced by H_2_O_2_ was dose dependent (Figure 2A upper panel). We selected H_2_O_2_ at 0.5 mM as our test concentration because cataracts formed at 0.3 mM H_2_O_2_ were less stable, and some cataracts spontaneously reversed. In comparison, 1 mM H_2_O_2_ produced severe cataracts that also severely disrupted lens morphology. In order to determine the effect of H_2_O_2_ on lenses with different genotypes, we normalized opacity intensity by subtracting the opacity intensity before H_2_O_2_ treatment because the lenses of Cx50KO and Cx46KO mice already had a certain level of lens opacity compared to WT.^20^^,^^21^^,^^22^^,^^23^ We found that after treatment with the same concentration of H_2_O_2_, the Cx50KO lens had the largest increase in opacity intensity compared to Cx46KO and WT lenses, and the WT lens had the least increase in opacity (Figure 2A). Similarly, we used different energy levels of UVB to induce cataracts in WT, Cx50KO, and Cx46KO mouse lenses. In the presence of UVB, unstable cataracts were found under 2J/cm^2^ and severe cataracts at 20J/cm^2^, thereby 10J/cm^2^ was selected for our study. We also found that the lens opacity increased the most in Cx50KO, followed by Cx46KO, and the least in WT after treatment under identical energy levels of UVB (Figure 2B). These results demonstrated that Cx50 and Cx46 are involved in preventing cataract formation induced by H_2_O_2_ and UVB, and Cx50 appears to play a major role.

### PKA activators reduced H_2_O_2_- or UVB-induced cataracts in WT, Cx50KO, and Cx46KO, but not in dKO lenses

Our previous study has shown that PKA activators, 8-Br-cAMP (1 mM) and forskolin (10 μM), enhance both GJs and HCs functions in chicken embryonic fibroblast (CEF) cells.^26^ To further verify their effects in the lens organ, we first used the WT mouse lens to determine the optimal concentration of forskolin and 8-Br-cAMP. We found that both forskolin and 8-Br-cAMP alleviated cataracts caused by H_2_O_2_ in isolated 4-months-old mouse lens. The effective concentration of forskolin was 50 μM (Figure S1A), and the effective concentration of 8-Br-cAMP was 2 mM (Figure S1B).

**Figure 3:**
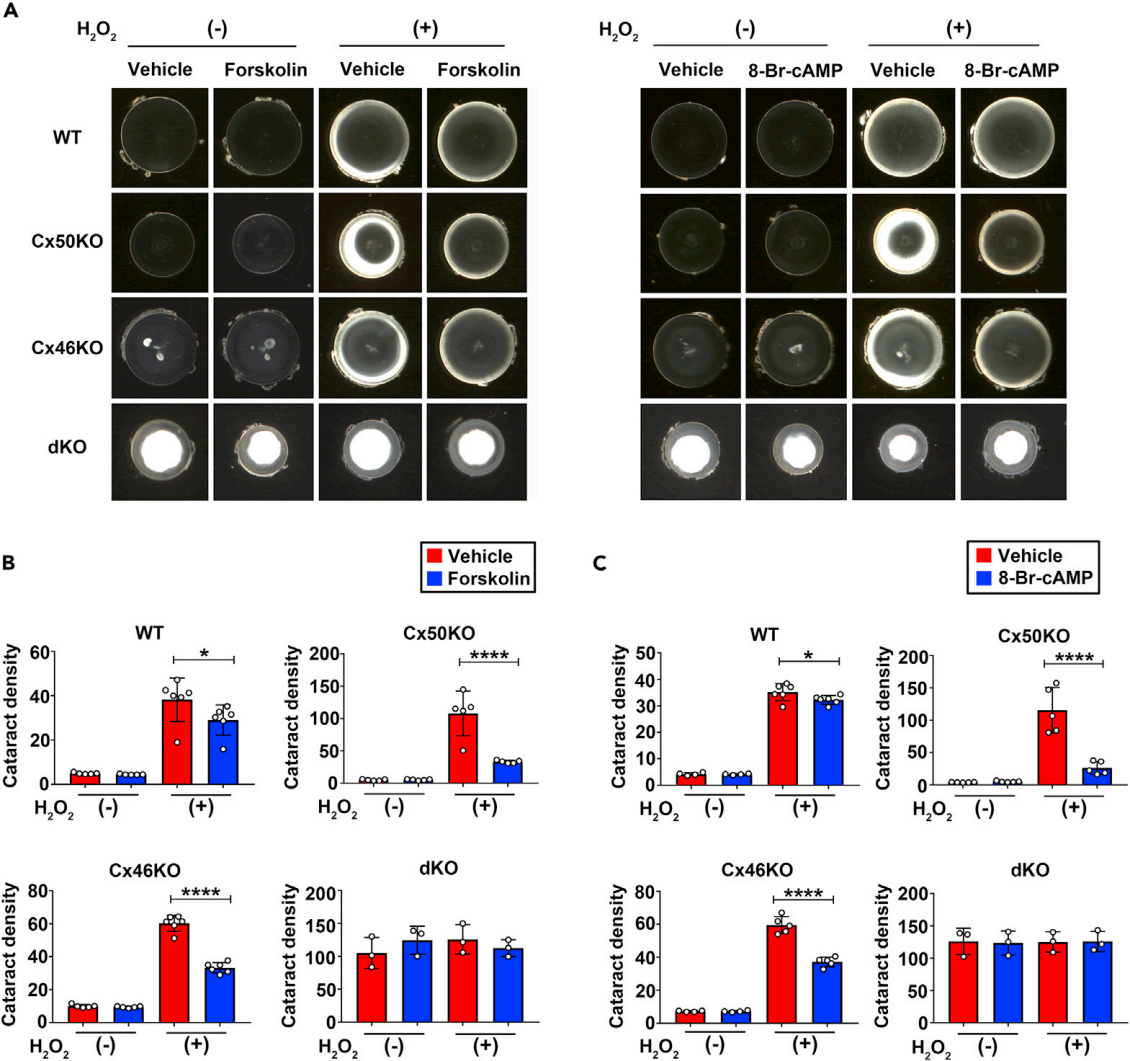
PKA activation reduces cataracts induced by H_2_O_2_ in lens of WT, Cx50KO, and Cx46KO, but has no effect on Cx46/Cx50 dKO (A–C) The lenses of WT, Cx50KO, Cx46KO, or dKO mice were kept transparent in culture media for 24 h with 5% CO_2_ at 37°C before being treated with or without 0.5 mM H_2_O_2_ for 4 h, followed by treatment with 50 μM Forskolin or vehicle (DMSO) (left panel) or 2 mM 8-Br-cAMP or vehicle (dH_2_O) as the control group (right panel). The opacity intensity of WT, Cx50KO, Cx46KO, and dKO in forskolin (B) or 8-Br-cAMP (C)-treated groups was measured by NIH ImageJ software. The data are presented as the mean ± SEM. (n ≥ 3). ∗, p < 0.05; ∗∗, p < 0.01; ∗∗∗, p < 0.001; ∗∗∗∗, p < 0.0001 (two-way ANOVA).

Based on our previous study,^26^ we used WT and Cx46KO mouse lens to test our hypothesis that the PKA activator enhanced Cx50 channel function and reduced cataract formation. We expected that PKA activators would be ineffective in Cx50KO. Interestingly, PKA activator, forskolin at 50 μM, not only reduced H_2_O_2_-induced cataract formation in the Cx46KO lens but also in the Cx50KO lens. However, the forskolin effect on improving lens opacity intensity was not shown in Cx46/Cx50 dKO mouse lenses (Figures 3A and 3B). Another PKA activator, 8-Br-cAMP at 2 mM has a similar effect as forskolin on the reduction of lens opacity induced by H_2_O_2_ in WT and Cx50 and Cx46 single KO lenses (Figure 3C), and such reduction was also not seen in dKO lens (Figures 3A and 3C). These results suggest that Cx50 and Cx46 likely mediate the effect of PKA activators.

**Figure 4:**
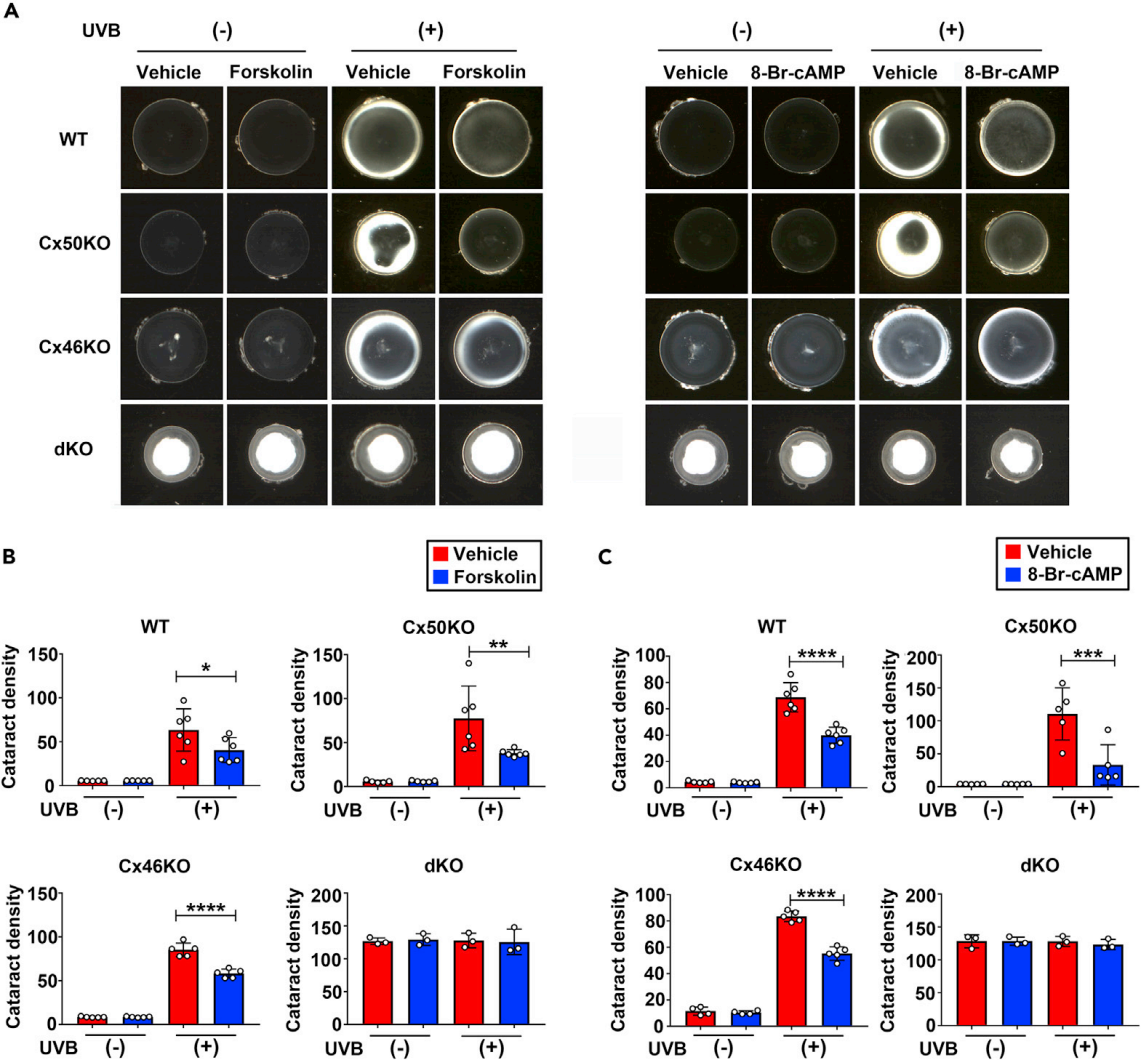
Activation of PKA reduces cataracts induced by UVB in lens of WT, Cx50KO, and Cx46KO, but has no effect on Cx46/Cx50 dKO (A–C) The lenses of WT, Cx50KO, Cx46KO, or dKO mice were kept transparent in culture media for 24 h with 5% CO_2_ at 37°C before being treated with or without 10 J/cm^2^ UVB radiation 4 h, followed by treatment with 50 μM forskolin or vehicle (DMSO) (left panel) or 2 mM 8-Br-cAMP or vehicle (dH_2_O) (right panel). The opacity intensity of WT, Cx50KO, Cx46KO, and dKO in forskolin (B) or 8-Br-cAMP (C)-treated groups was measured by NIH ImageJ software. The data are presented as the mean ± SEM. (n ≥ 3). ∗, p < 0.05; ∗∗, p < 0.01; ∗∗∗, p < 0.001; ∗∗∗∗, p < 0.0001 (two-way ANOVA).

**Figure 5:**
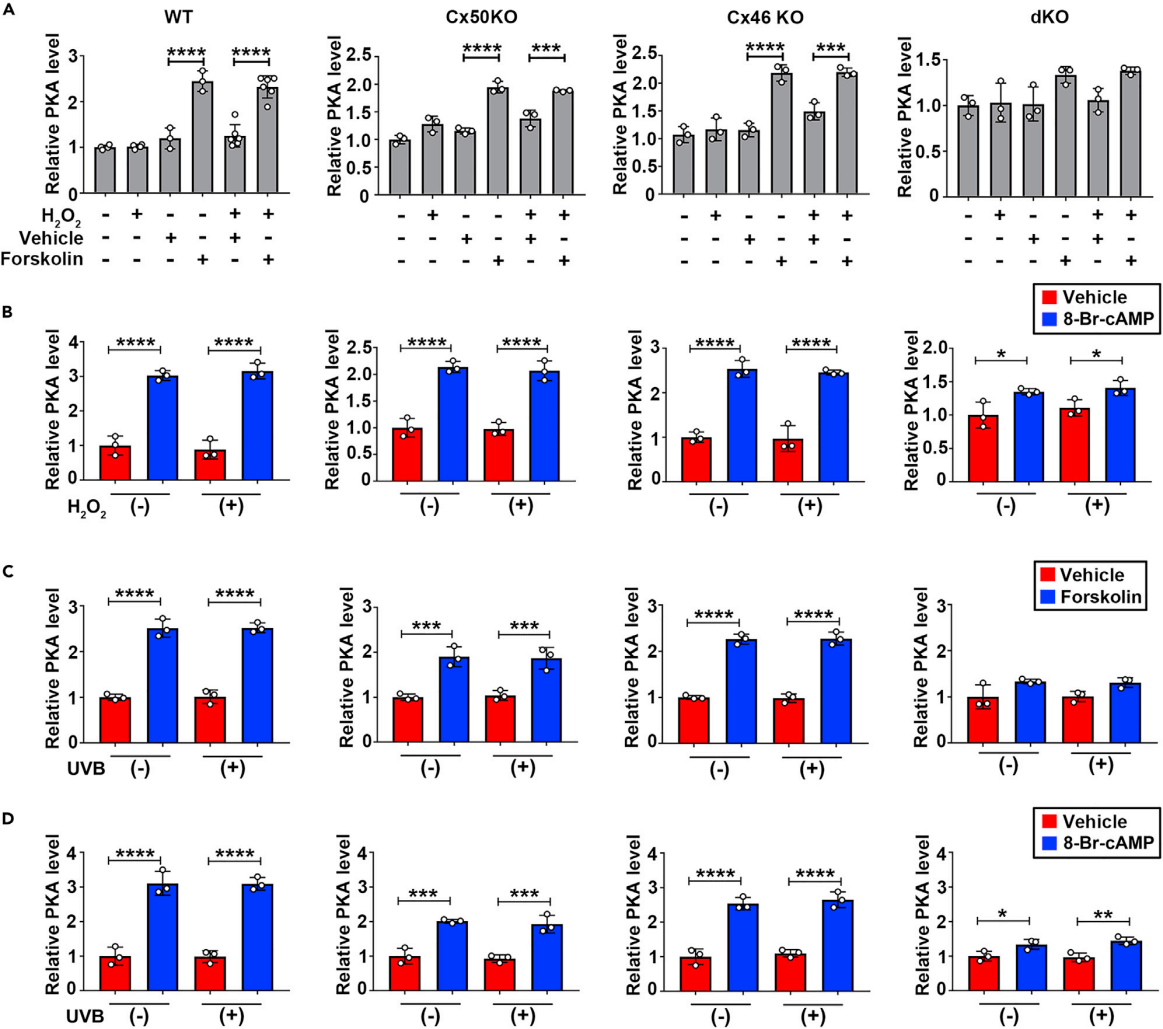
PKA activators increase PKA levels in the lens of WT, Cx50KO, Cx46 KO, and Cx46/Cx50 dKO in the absence or presence of H_2_O_2_ and UVB (A–C) The lenses of WT, Cx50KO, Cx46KO, and dKO mice PKA levels were tested and quantified by Invitrogen PKA (Protein Kinase A) Colorimetric Activity Kit after lens were treated with or without 50 μM forskolin in the absence or presence of 0.5 mM H_2_O_2._ (One-way ANOVA) (B) PKA levels were tested and quantified after lens was treated with or without 2 mM 8-Br-cAMP in the absence or presence of 0.5 mM H_2_O_2._ (C) PKA levels were tested and quantified after the lens were treated with or without 50 μM forskolin in the absence or presence of 10 J/cm^2^ UVB radiation. (D) PKA levels were tested and quantified after lens was treated with or without 2 mM 8-Br-cAMP in the absence or presence of 10J/cm^2^ UVB radiation. The data are presented as the mean ± SEM. (n ≥ 3). ∗, p < 0.05; ∗∗, p < 0.01; ∗∗∗, p < 0.001; ∗∗∗∗, p < 0.0001. (two-way ANOVA for B-D).

UVB radiation (280–315 nm) exposure that elevates oxidative stress levels in the lens is one of the leading causes of age-related cataracts.^6^^,^^29^ We investigated whether PKA activators (forskolin and 8-Br-cAMP) could alleviate UVB-induced cataracts. We applied 50 μM forskolin or 1 mM 8-Br-cAMP to WT, Cx50KO, Cx46KO, and dKO lenses after UVB irradiation and captured the images 24 h after treatment (Figure 4A). Similar to the results obtained by the H_2_O_2_ treatment, forskolin alleviated UVB-induced cataracts in WT, Cx50KO, and Cx46KO mouse lenses, whereas there was no effect in lens opacity intensity in dKO lens in response to forskolin (Figure 4B) and 8-Br-cAMP (Figure 4C). We also determined PKA activation in WT and KO lenses. Forskolin and 8-Br-cAMP increased PKA activation in WT, Cx50KO, and Cx46KO lenses with or without H_2_O_2_ or UVB (Figures 5A–5D, ). However, similar to what we observed for lens opacity, the extent of PKA activation by forskolin and 8-Br-cAMP in dKO lens was less than WT and Cx50 and Cx46 single KO. Together, these results suggest that the effect of PKA activators on preserving lens transparency against oxidative stress (H_2_O_2_ and UVB) is mediated by Cx50 and Cx46, while the protective effect with deletion of either Cx50 or Cx46 could be caused by the compensatory mechanism of these two Cxs.

### PKA activators increase Cx46 homomeric and Cx46 and Cx50 heteromeric GJs coupling and HCs activities

**Figure 6:**
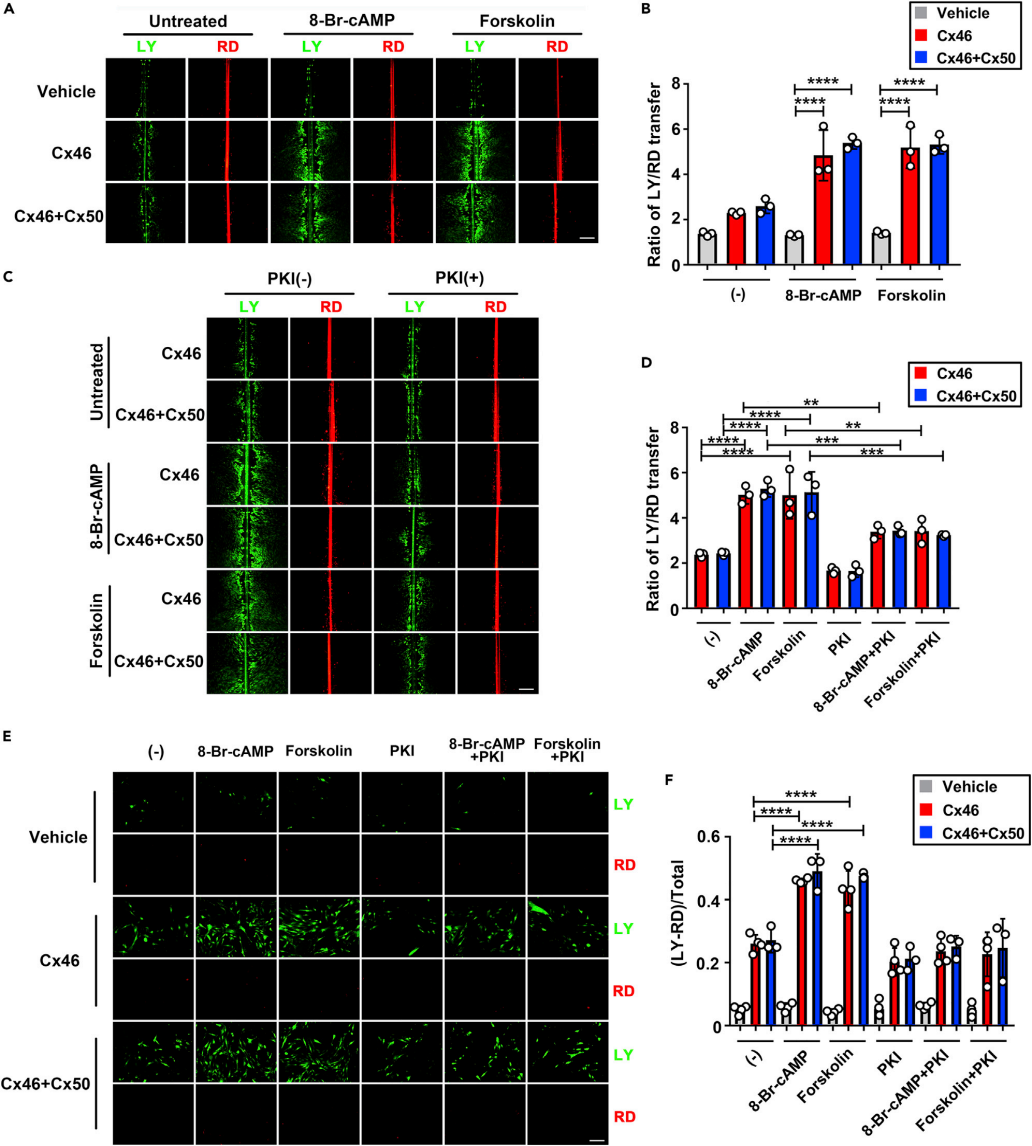
PKA activators increase GJ coupling and HC activities in Cx46 or Cx46 and Cx50 co-expressed cells, and this increase is alleviated by a PKA inhibitor (A–D) CEF cells were infected with high-titer RCAS(A) retroviral vehicle (V) or recombinant RCAS(A) retroviruses containing Cx46 or co-infected with RCAS(A) containing Cx46 and Cx50, and cells were grown to confluence to maximize cell-cell contact. (A and B) Cells were treated with or without PKA activator: 8-Br-cAMP (1 mM) or forskolin (10 μM) for 2 h before scrape loading dye transfer assay using LY (green) as a tracer for GJs coupling and RD (red) as a tracer for originally dye-loaded cells. The extent of dye transfer was measured as the ratio of LY-labeled cells to that of RD-labeled cells. The data are presented as the mean ± SEM. (n = 3). ∗∗∗∗, p < 0.0001. (two-way ANOVA). (C and D) Cells were treated or not with 8-BrcAMP (1 mM) or forskolin (10 μM), with or without PKI (0.4 μM) for 2 h before scrape loading dye transfer assay. Scale bar: 50 μm. The extent of dye transfer was measured as the ratio of LY-stained cells to that of RD-labeled cells. The data are presented as the mean ± SEM. (n = 3). ∗∗, p < 0.01; ∗∗∗, p < 0.001; ∗∗∗∗, p < 0.0001 (two-way ANOVA). (E and F) CEF cells infected with high-titer retrovirus RCAS(A) vehicle (V) or recombinant RCAS(A) containing Cx46 or co-infected with RCAS(A) containing Cx46 and Cx50 were cultured at low cell density with no physical contact. Cells were treated or not with 8-BrcAMP (1 mM) or forskolin (10 μM) with or without PKI (0.4 μM) for 2 h before FFSS at 1 dyne/cm^2^ for 30 min and followed by dye uptake assay with LY and RD. At least three microphotographs of fluorescence fields were captured. Scale bar: 50 μm. The LY uptake percentage was quantified by subtracting LY/RD double-positive cells from LY-positive cells. The data are presented as the mean ± SEM. (n = 3). ∗∗∗∗, p < 0.0001 (two-way ANOVA).

We have previously shown that PKA activation enhanced both Cx50 GJs and HCs functions.^26^ Here, we showed that PKA activators surprisingly alleviated the H_2_O_2_-induced opacity in the Cx50KO lenses, hence hypothesizing that Cx46 in Cx50KO lens may compensate for the role of Cx50. Therefore, we determined the effect of PKA activators on the function of Cx46 and Cx46 + Cx50 GJs and HCs activities. CEF cells expressing exogenous Cx46, both Cx46 and Cx50, or vehicle (RCAS(A)) via retroviral infection were pretreated with the PKA activator 8-Br-cAMP or forskolin, and GJs coupling was evaluated by scrape loading dye transfer assay. To determine the effective concentration, we used different concentrations (0 μM, 1 μM, 10 μM, and 50 μM) of forskolin in the vehicle, with the Cx46 or Cx46 + Cx50 groups (Figure S2A). We found that the effective dose of forskolin *in vitro* is 10 μM (Figure S2B), the same dose as we previously reported.^26^ We then performed a scrape loading dye transfer assay in CEF cells expressing Cx46, both Cx46 and Cx50, or vehicle control in the presence of PKA activator: 8-Br-cAMP (1 mM) or forskolin (10 μM) (Figure 6A). Both 8-Br-cAMP and forskolin significantly enhanced the GJs coupling in cells expressing Cx46 and both Cx46 and Cx50, but not in vehicle control cells (Figure 6B). In addition, we found that the increase induced by PKA activators was abolished by a specific PKA inhibitor, PKI (Figures 6C and 6D).

Previous studies have shown that the activation of HCs by fluid flow shear stress (FFSS) serves as a portal for the influx of glucose and GSH into cortical fiber cells,^30^ and we have previously demonstrated the activation of Cx50 HCs by PKA.^26^ Here, we determined whether PKA enhanced Cx46 homomeric or Cx46/Cx50 heteromeric HCs function by dye uptake assay. Cells pretreated with PKA activators were subjected to FFSS, and Lucifer yellow/rhodamine dextran dye uptake assay was performed. A comparable increase of dye uptake was observed in CEF cells expressing Cx46 or both Cx46 and Cx50 (Figure 6E), and this increase was significantly attenuated by PKI. The vehicle control cells were unresponsive to PKA and PKI (Figure 6F). These results suggest that PKA activators increase both Cx46 homomeric and Cx46/Cx50 heteromeric HCs opening.

### Reduction of anti-oxidative stress gene expression in Cx-deleted lens

**Figure 7:**
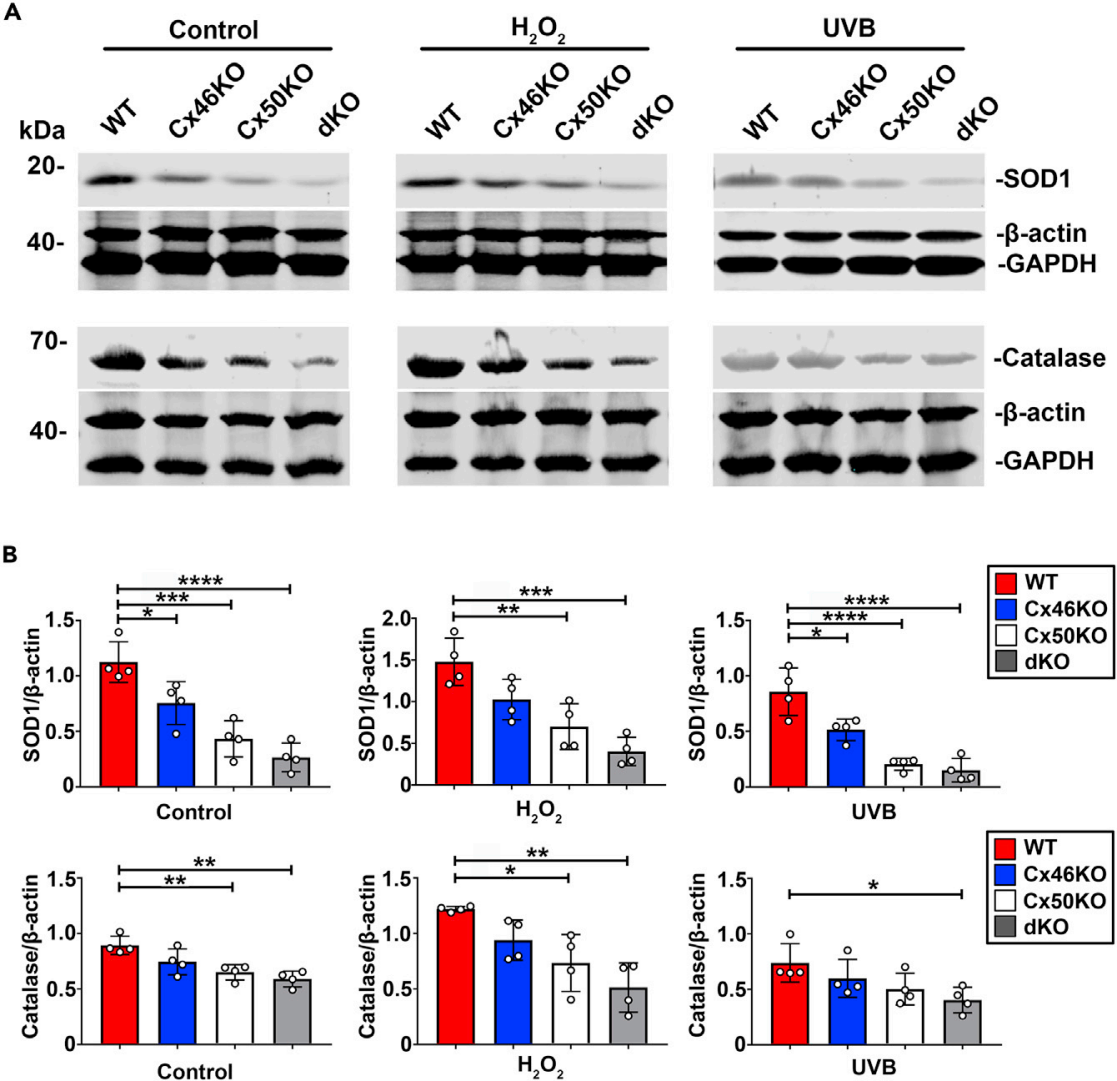
Reduction of anti-oxidative stress gene expression in Cx-deleted lens (A) The protein extracts of the lenses from WT, Cx46KO, Cx50KO, and dKO mice in untreated control, H_2_O_2_-, or UVB-treated group were immunoblotted with anti-SOD1, catalase, β-actin, or GAPDH antibody. (B) The intensity of the bands was quantified using Image Studio Lite Ver 5.2 software, and the ratio of SOD1 to β-actin and catalase to β-actin was quantified. The data are presented as the mean ± SEM. (n = 4). ∗, p < 0.05; ∗∗, p < 0.01; ∗∗∗, p < 0.001; ∗∗∗∗, p < 0.0001 (One-way ANOVA).

Western blotting detected the expression of anti-oxidative proteins, SOD1 (Figure S3A) and CAT (Figure S3B), in four-month-old WT, Cx46KO, Cx50KO, and dKO lenses. The level of SOD1 and CAT was the highest in WT, followed by Cx46KO, then Cx50KO, and the lowest in dKO. Immunostaining with antibodies for oxidative stress markers, SOD1 and CAT, was used to evaluate the oxidative stress level with or without treatment of H_2_O_2_ or UVB. In Cx-deleted lens, reduced SOD1 and CAT levels were detected across various regions of the lens with or without H_2_O_2_ or UVB, especially in dKO lens, and little expression was detected in the nucleus region of all genotype mice (Figures S4A and S4B). The protein expression levels were detected by western blots (Figure 7A). Similar to the untreated group, in both the H_2_O_2_-treated and UVB-treated groups, the SOD1 and CAT expression levels were highest in WT, followed by Cx46KO, then Cx50KO, and the lowest in dKO. Interestingly, there was an opposite response of SOD1 and CAT to the stimuli; increased by H_2_O_2_ while decreased by UVB (Figure 7B). This responsive pattern of SOD1 appears to be consistent with our previous study in HLE-B3 lens epithelial cells.^31^

### PKA activation decreases ROS level in WT, Cx46, and Cx50 KO, but not in Cx46/Cx50 dKO lens

**Figure 8:**
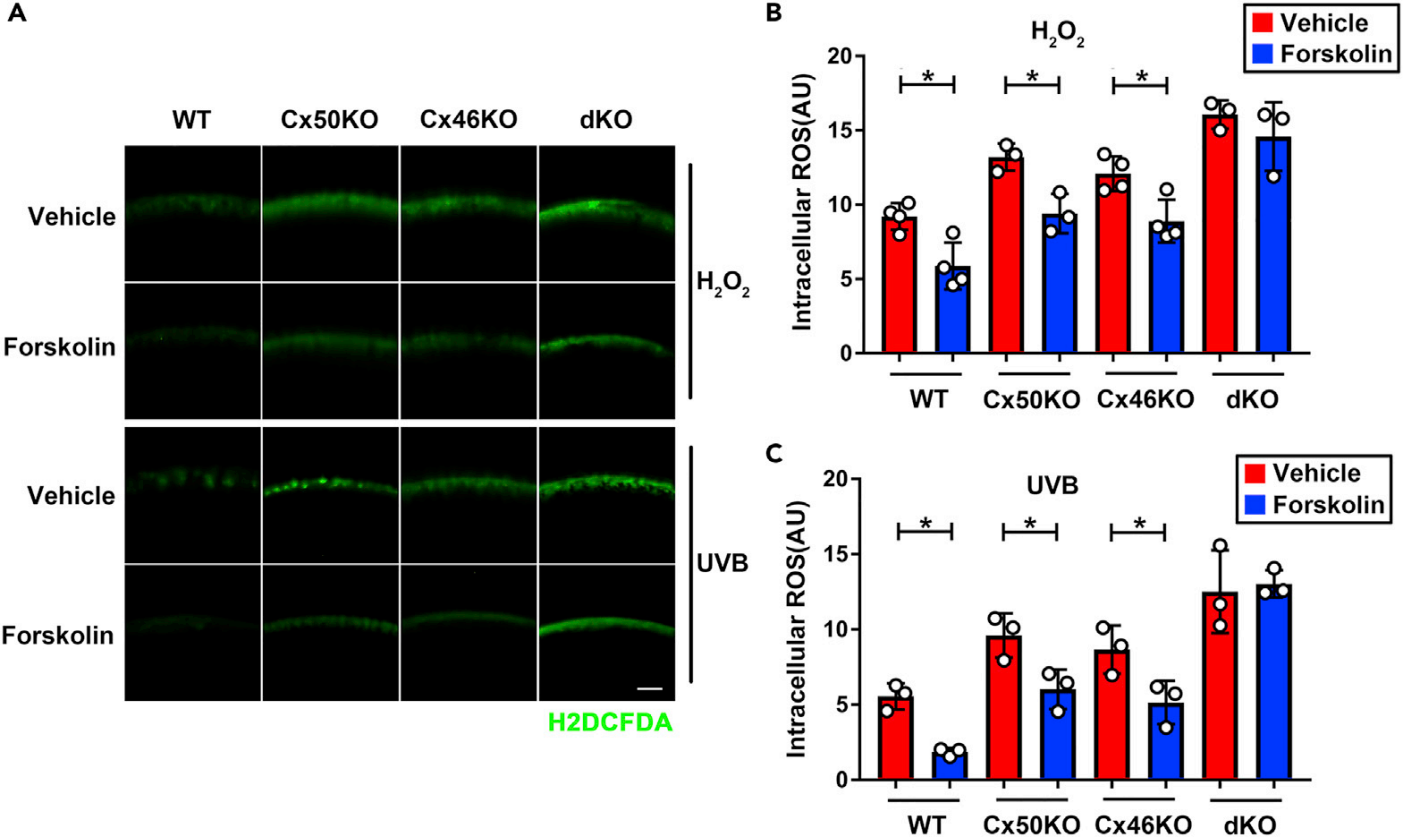
PKA activation decreases ROS levels in WT, Cx50, and Cx46 KO, but not in Cx46/Cx50 dKO lens (A–C) The lenses of WT, Cx50KO, Cx46KO, and dKO mice were kept transparent in culture media for 24 h at 37°C before being treated with 0.5 mM H_2_O_2_ (upper panel) or 10 J/cm^2^ UVB (lower panel) for 4 h, and followed by treatment with 50 μM forskolin, or vehicle (DMSO) for 2 h. Intracellular ROS was measured by incubating lens with 10 μM Carbxyl-H2DCFDA for 30 min. Scale bar: 100 μm. H_2_O_2_-induced (B) or UVB-induced (C) intracellular ROS level was quantified by using NIH ImageJ software. The data are presented as the mean ± SEM. (n ≥ 3). ∗p < 0.05, ∗∗p < 0.01, ∗∗∗p < 0.001, ∗∗∗∗p < 0.0001 (two-way ANOVA).

Persistently elevated intracellular ROS causes oxidative damage to lens cells, leading to cataracts.^5^^,^^32^ We determined the intracellular ROS levels after H_2_O_2_ treatment with or without forskolin and concurrently the lens opacity intensity at various time periods (Figure S5A). Our time-course experiments showed that the intracellular ROS levels of the non-forskolin-treated group peaked at 2 h after 4-h H_2_O_2_ treatment, and forskolin significantly reduced ROS levels at this time point. (Figure S5B). In contrast, WT lenses started to develop lens opacity after 4 h of H_2_O_2_ treatment in WT lens, and there was no difference between forskolin-treated and non-treated control lens until 20 h of treatment with forskolin (Figure S5C). Similar elevation of intracellular ROS level at 2 h after 4-h H_2_O_2_ treatment or UVB was observed in Cx50 or Cx46 single KO and dKO, and the most significant increase was observed in dKO lens (Figure 8A). Forskolin treatment significantly reduced the opacity in WT and Cx50, and Cx46 single KO lens, but not in dKO lens after H_2_O_2_ (Figure 8B) or UVB (Figure 8C). These results suggest that lens deficient of both Cx50 and Cx46 is more susceptible to oxidative insults and cataract formation, and Cx50 and/or Cx46 are indispensable for the action of PKA activators.

## Discussion

Unlike lens epithelial cells, fiber cells are metabolically inactive and never renew throughout an animal’s life cycle, so dysfunction of the detoxification system and accumulation of ROS makes lens fiber cells more susceptible to oxidation during aging.^5^^,^^33^ Avascular lenses require the constant maintenance of lens redox homeostasis and transparency through peripheral fluid diffusion and/or the transport of nutrients or antioxidants (such as GSH) through a transporter and channel network-based microcirculatory system.^9^^,^^34^^,^^35^ GSH is an important antioxidant in the eye, where it is used to detoxify damaging oxidants.^36^ Under UVB or other oxidative stress in aged eyes, GSH level at both the intracellular and extracellular environments of lens fibers could be affected. In addition to extracellular GSH contributed from the aqueous humor, lens epithelial cells also synthesize GSH *in situ*. Under stress conditions and aging, the GSH production by epithelia cells could be affected as well. It is expected that the extracellular GSH level is lower than that in normal lens.^37^^,^^38^^,^^39^ However, there is also a study showing that the level of GSH in the aqueous humor of patients with cataract is actually several times higher than that of normal aqueous humor.^40^ Nevertheless, our research showed that increased HC activity in lens fibers by activated PKA will likely maximize the uptake of GSH to protect the lens fiber cells against these insults in lens. Increased GJs between fiber cells will aid in transferring GSH to inner fiber cells. Here, we cannot exclude the involvement of other alternative pathway(s). In addition to being secreted into the anterior aqueous humor by the ciliary body,^41^ exogenous GSH can also come from the vitreous body. GSH in the vitreous body could enter the lens by passive diffusion, and it is speculated that it may be due to connexin HCs.^42^ In mammalian lens fiber cells, Cx50 and Cx46 are the major components in the microcirculatory system.^15^^,^^43^ Moreover, our earlier evidence has suggested that PKA phosphorylates at Ser-395, a highly conserved residue across species including human, mouse, and chicken. This phosphorylation increases Cx50 GJs and HCs activities by increasing conductivity or gating properties of Cx50 channels.^26^ But it remained unknown if upregulation of lens connexin expression and channel activity by PKA could reduce oxidative stress in fiber cells and alleviate cataract formation. In this study, our data showed that PKA activators reduced intracellular ROS and protected lens fiber cells against oxidative stress-induced cell death. Importantly, we found PKA activators alleviated cataract formation only in WT and Cx50 and Cx46 single KO, but not in dKO lens. The increased GJ activity by PKA depends on phosphorylation level of connexins. Given that connexins in lens fibers have a very long half-life and lens fibers are less metabolically active associated with low phosphatase activities, PKA is likely to have a long-lasting effect on connexin channel activation. Our results unveil the new potential therapeutic approach by treating cataracts through activation of PKA, and this mechanism is mediated via the enhanced Cx50 and Cx46 functions. Thus, intermittent treatment of PKA activators could potentially alleviate cataracts in therapeutic application.

An earlier study by Calvin et al.^44^ indicated that H-89 (an inhibitor of PKA) could cause severe disturbances of lens electrolytes that possibly introduced large cortical cataracts. Walsh and Patterson reported that PKA activators, forskolin or 8-Br-cAMP, increased the equatorial current of the lens.^45^ We showed in our earlier study that PKA directly phosphorylates Cx50 and increases GJs and HCs function.^26^ We also showed that Cx HCs activated by FFSS act as portals to deliver GSH into lens cells.^30^ GSH, an antioxidant, could easily pass through the pores of Cx channels.^46^ In addition, studies have shown that the changes to redox potential triggered by oxidative stress induce the opening of GJs and HCs.^14^^,^^47^ However, the lipid peroxidation by oxidative stress and inflammation induces the carbonylation of Cx46 in the lens and reduces HC activity in *Xenopus* oocytes.^48^ Another earlier study of ours showed that Cx50 HCs activated by H_2_O_2_ mediate GSH transport and protect lens fiber cells from oxidative stress,^25^ and mechanically activated Cx HCs and GSH transport can reduce H_2_O_2_- and UVB-induced intracellular ROS, thereby further mitigating apoptosis and death.^30^ In this study, we found that forskolin reduced apoptosis and necrosis in primary lens cells when exogenously expressing WT Cx50; however, that reduction was ablated in Cx50H156N-expressed cells with impaired HCs. The data confirmed PKA effect on protecting lens cells against oxidative stress is through HCs, and this mechanism is likely through the reported role of HCs in antioxidant transport. These studies further suggest that Cx HCs could serve as a potent target in fiber cells to combat oxidative damage^30^ and PKA activators would maximize the function of HCs in antioxidant delivery and lens protection.

Oxidative stress is one of the major risk factors for cataracts, and elevated oxidant H_2_O_2_ level is seen in the lens and aqueous humor of patients with cataract.^6^ Studies also show a higher incidence of cataracts in environments with relatively high UV index,^49^^,^^50^ and continued exposure to UV radiation and elevated H_2_O_2_ levels in the surrounding environment are two major sources of oxidative stress in the lens.^5^ In this study, we used the two well-characterized means, H_2_O_2_ and UV radiation, to elevate oxidative stress and generate opacity in mouse lens. H_2_O_2_ is a fast-acting oxidizing reagent,^27^ while the UVB, one of the three UVRs with a wavelength of 280–315 nm, is responsible for photochemical reactions that damage the lens by generating ROS.^49^^,^^51^^,^^52^ To delineate the therapeutic potential of the PKA inhibitors, we first induced lens opacity with H_2_O_2_ and UVB and then treated with PKA activators. We found that PKA activators can extenuate cataracts in WT, possibly through enhanced Cx50 GJs and HCs by PKA activators, as we have previously shown.^26^ Surprisingly, PKA activators also alleviated H_2_O_2_- or UVB-induced cataracts in Cx50KO lens. In addition to GJs, Cx46 also forms functional HCs on the nonjunctional membrane of fiber cells.^16^ We therefore examined the responsiveness of Cx46 KO to PKA inhibitors and found the significant reduction of opacity induced by H_2_O_2_ and UVB. These data indicate that Cx46 channels like Cx50’s may be positively regulated by PKA activation.

We decided both GJs and HCs formed by homomeric Cx46 or heteromeric Cx46/Cx50. The HCs formed by Cx46 and Cx50 are mechanosensitive, and lens fiber cells are subjected to mechanical stimulation due to FFSS generated by lens microcirculation.^30^^,^^53^ The shear stress level we estimated is about 1 dyne/cm^2^, and we used this level of FFSS to induce HC opening.^30^ Indeed, we found that gap junctions and HCs, both Cx46 and also Cx46/Cx50, like those of Cx50, were enhanced by PKA activators. The upregulation of GJs and HCs function by PKA activators was abolished by a specific PKA inhibitor PKI, further validating the specific effect of PKA activation. Because PKI only partially inhibited GJ communication activated by PKA in our dye transfer assay, we did not use this inhibitor to assess cataract formation. This enhancement provides an explanation regarding the improvement of lens opacity by PKA activators even in the lens with the deletion of either Cx50 or Cx46 gene. Evidence from previous studies has shown that knockin of Cx46 partially rescues Cx50 KO lens fiber defects.^54^ Furthermore, both Cx50 and Cx46 in the lens are significantly downregulated with age in humans and mice,^55^ implying the increased vulnerability of the aging lens to cataracts. The responsiveness to PKA activation in these single KO mouse models suggests that Cx50 and Cx46 complement each other in protecting the lens against oxidative insults.

We showed that PKA inhibitors failed to alleviate lens opacity when both Cx46 and Cx50 genes were deleted in dKO lens. The data further confirm that Cx50 and Cx46 are the targets of PKA, and enhanced GJ’s and HC’s protect lens from H_2_O_2_ and UVB-induced opacity. PKA activation by forskolin and 8-Br-cAMP was also lower in dKO lens. This could be caused by impaired GJs in lens fiber cells since lens GJs play an important role in transferring cAMP between the cells.^56^ We also observed that H_2_O_2_ and UVB did not lead to a further increase in lens opacity in dKO. This could be partially explained by the severe cataracts developed in dKO mice as well as distorted lens morphology.^23^

We found that after treatment with the same concentration of H_2_O_2_ or intensity of UVB, the Cx50KO lens had a greater increase of lens opacity than Cx46KO, implying Cx50 may have a higher capacity in reducing oxidative stress in lens, likely through the regulation of expression of anti-oxidative stress genes. Studies show that H_2_O_2_ can regulate the redox state by controlling the activity of antioxidant enzymes or the transcription of genes encoding antioxidant enzymes.^27^^,^^57^ Indeed, Cx50KO exhibited a lower expression level of two major antioxidant enzymes, SOD1 and CAT, than WT and Cx46KO. As expected, the lowest level of expression was detected in dKO lens deficient of both Cx46 and Cx50. It is not certain why knocking out connexin causes the downregulation of antioxidant enzymes. The change of enzyme expression could be an adaptive response in response to intracellular ROS level.^58^^,^^59^ We have observed a similar downregulation of antioxidant enzymes in the lens of heterozygous Cx43 knockout mice.^31^ The reduction of these enzymes associated with compromised antioxidant capability is a likely underlying cause of increased oxidative stress and cataracts in connexin KO lenses. The anti-oxidative roles of Cx46 and Cx50 in lens fiber were also manifested by previously reported knockin mouse models, Cx46fs380 and Cx50D47A, the lenses of both mouse lines have a more oxidizing environment than wild-type lenses.^60^ The expression of antioxidant enzymes in the lens fiber cells increased after H_2_O_2_ treatment. Interestingly, we observed an opposite response of antioxidant enzymes to UVB as they decreased slightly after UVB treatment. This response is consistent with our previous findings in the lens epithelium.^31^ Previous studies have shown that transient increases in intracellular H_2_O_2_ in lens epithelial cells may upregulate transcription factors that upregulate antioxidant gene expression.^57^ While under UVB, the photosensitive molecules react with oxygen to produce the superoxide anion (O2^·-^);^61^ therefore, the main difference here is that UVB induces the cells to directly produce O2^·-^ instead of H_2_O_2_. In addition to generating ROS, UVB also causes direct DNA damage through photo-oxidation,^29^ which may lead to a decrease in gene transcription, which in turn leads to decreased expression of antioxidant enzymes. Together, our study demonstrated that PKA activation protects lens fiber cells against oxidative stress-induced cell death, lens opacity, oxidative stress, and downregulation of antioxidant enzymes. The protective role of PKA activation in the lens is fulfilled through its effect on the enhancement of lens fiber Cx50 and Cx46 expression and HC function. The outcome of this study is likely to provide a novel therapeutic approach for cataract prevention and treatment.

### Limitations of the study

We acknowledge certain limitation in our study. In this study, we used *ex vivo* experiments to demonstrate that PKA activators protect lens from oxidative stress-induced cell death, lens opacity, oxidative stress, and downregulation of antioxidant enzymes. We did not perform *in vivo* experiments in mice to determine the protective effects of PKA activators due to limitation of the models.

## STAR★Methods

### Key resources table

**Table:** 

REAGENT or RESOURCE	SOURCE	IDENTIFIER
**Antibodies**		

Rabbit anti-SOD1	Santa Cruz Biotechnology, Dallas, Texas	Cat# sc-11407; RRID:AB_2193779
Rabbit anti-catalase	Santa Cruz Biotechnology, Dallas, Texas	Cat# sc-50508; RRID:AB_2275410
Monoclonal mouse anti-β-actin	Thermo Fisher Scientific	Cat# MA5-15739; RRID:AB_10979409
Monoclonal mouse anti-GAPDH	Thermo Fisher Scientific	Cat# AM4300; RRID:AB_2536381

**Bacterial and virus strains**		

Recombinant RCAS(A) DNA constructs and high-titer retroviruses	Jiang and Goodenough, 1998^65^	University of Texas Health Science Center at San Antonio
Cx50H156N	Banks et al., 2007^66^	University of Texas Health Science Center at San Antonio

**Biological samples**		

Fertilized white leghorn chicken eggs	Texas A&M Agriculture & Policy Science	Fertile egg

**Chemicals, peptides, and recombinant proteins**		

Protease Inhibitor Cocktail	Thermo Fisher Scientific	Cat# 5892970001
Lucifer yellow	Thermo Fisher Scientific	Cat# L453
Rhodamine dextran	Thermo Fisher Scientific	Cat# T669
Dimethyl sulfoxide	Thermo Fisher Scientific	Cat# MT-25950CQC
Forskolin	Thermo Fisher Scientific	Cat# 34-427-010MG
Carboxy H2DCFDA	Thermo Fisher Scientific	Cat# C400
Paraformaldehyde	Thermo Fisher Scientific	Cat# 15710
Dulbecco’s modified Eagle’s medium (DMEM, high glucose)	Thermo Fisher Scientific	Cat# 11965118
Hydrogen peroxide, 30%	Thermo Fisher Scientific	Cat# H325-100
Chicken serum	Thermo Fisher Scientific	Cat# 16-110-082
TrypLE Express enzyme	Thermo Fisher Scientific	Cat# 12605–028
Medium 199	Thermo Fisher Scientific	Cat# 11-150-059
Phenol red-free medium 199	Thermo Fisher Scientific	Cat# 11-043-023
Penicillin-streptomycin	Thermo Fisher Scientific	Cat# MT30001CI
8-Br-cAMP	Thermo Fisher Scientific	Cat# NC1183334
Andwin ScientificTissue-Tek™ CRYO-OCT Compound	Thermo Fisher Scientific	Cat# 14-373-65
Thermo Scientific™ Pierce™ DAPI Nuclear Counterstain	Thermo Fisher Scientific	Cat# PI-62247

**Critical commercial assays**		

Dead Cell Apoptosis Kit	Biolegend	Cat# 640914
PKA Colorimetric Activity Kit	Thermo Fisher Scientific	Cat# EIAPKA

**Experimental models: Cell lines**		

Chicken embryonic fibroblast (CEF)	Laboratory of Dr. Jean Jiang	University of Texas Health Science center at San Antonio
Chick lens primary cell	Jiang et al., 1993^63^	University of Texas Health Science center at San Antonio

**Experimental models: Organisms/strains**		

Cx50 knockout (Cx50^−/−^) mouse; C57BL/6	White et al., 1998^22^	Stony Brook University
Cx46 knockout (Cx46^−/−^) mouse; C57BL/6	White et al., 1998^22^	Stony Brook University
Cx46 and Cx50 double knockout (Cx46^−/−^/Cx50^−/−^) mouse; C57BL/6	Gu et al., 2019^23^	University of Texas Health Science center at San Antonio

**Software and algorithms**		

Image Studio Lite	Image Studio Lite software	Image Studio Lite; RRID:SCR_013715
ImageJ	NIH ImageJ software	ImageJ; RRID: SCR_003070
GraphPad Prism 7 software	GraphPad software	GraphPad; RRID: SCR_000306

### Resource availability

#### Lead contact

Further information and requests should be directed to and will be fulfilled by the lead contact: Jean X. Jiang (jiangj@uthscsa.edu).

#### Materials availability

This study did not generate new animal models and reagents.

### Experimental model and subject details

#### Mice

Four months old, both male and females wild-type (WT), Cx50 knockout (KO), Cx46KO, and double (d) KO mice were used in the study. The breeding pairs of the Cx50 knockout and Cx46 knockout mouse strains were generously provided by Dr. Thomas White at Stony Brook University (Stony Brook, New York, USA). Mice were housed in a temperature-controlled room with a 12-hrs light/12-hrs dark cycle and under specific pathogen-free conditions in the Institutional Lab Animal Research facility at the University of Texas Health Science Center at San Antonio (UTHSCSA).

#### Cell culture

Fertilized white leghorn chicken eggs were obtained from Texas A&M Agriculture & Policy Science (Collage Station, TX, USA) and incubated for 11 days in a Sportsman Egg Incubator in preparation for CEF and lens primary cells. CEF cells were cultured in DMEM plus 10% fetal bovine serum (FBS), 2% chick serum, 1% PS, and 1% sodium pyruvate in an incubator supplied with 5% CO_2_ at 37°C. CEF cells were infected with the high-titer retroviruses on the second day of culture. After reaching 95% confluence, cells were digested with pre-warmed TrypLE Express enzyme and passaged. Chick lens primary cell culture was prepared by a modified protocol as previously described.^62^^,^^63^ We adhered to ARRIVE guidelines to guarantee reporting *in vivo* experiments properly. The protocol for using chick embryos was reviewed and approved by the Institutional Animal Care and Use Committee (IACUC) at UTHSCSA. Briefly, lenses were dissected from 11-day-old chick embryos, washed with TD-buffer (140 mM NaCl, 5 mM KCl, 0.7 mM Na_2_HPO_4_, 5 mM glucose, and 25 mM Tris-HCl, pH 7.4) twice, digested with 0.1% trypsin in TD-buffer at 37°C for 20 min, and homogenized properly in medium 199 plus 10% FBS and 1% PS. Living cells were counted and seeded at the density of 4×10^5^ cells/well or 2×10^6^ cells/dish in type I collagen-coated 12-well plates or 60-mm dishes, respectively. On the second day of culturing, lens primary cells were infected with high-titer retroviruses. Cells incubated at 37°C supplied with 5% CO_2_ were fed every two days. On day 4–5, lens epithelial cells reached confluence and began to differentiate to form fiber-like lentoid structures.

### Method details

#### Site-directed mutagenesis and high-titer recombinant RCAS(A) retrovirus preparation

Recombinant RCAS(A) DNA constructs and high-titer retroviruses were prepared as previously described.^64^^,^^65^ Briefly, a cDNA fragment containing chick Cx50 and Flag tag at Cx50 C-terminus were generated by PCR and constructed into an RCAS(A) vector. We have shown minimal interference of a Flag tag on connexin membrane distribution and channel activities.^65^ Based on the RCAS(A)-Cx50 DNA construct, we generated recombinant RCAS(A) containing a Cx50 site mutant: Cx50H156N as previously reported.^25^^,^^66^^,^^67^ High-titer recombinant retroviruses were generated (1×10^8^–5×10^8^ colony-forming units (cfu) per mL) by transfecting DNA constructs into chicken embryonic fibroblast (CEF) cells using Lipofectamine® following the manufacturer’s guidelines (ThermoFisher). Conditioned medium was collected and centrifugated to generate high-titer retroviral stocks.

#### Lenses tissue isolation and culturing

Eyeballs were collected from 4 months old mice after euthanization. An incision on the eyeball at the posterior side was made to remove the lens under a dissection microscope (SM-1TSW2-L6W-5M, AmScope). Isolated lenses were cultured in phenol red-free Medium 199 containing 10% FBS and 1% PS and incubated for 24 hrs with 5% CO_2_ at 37°C. The lenses were examined to ensure their intactness and transparency throughout the entire procedure before treatment. Microscopic images of lenses were acquired using a dissecting microscope (SM-1TSW2-L6W-5M, AmScope).

#### Cataractogenesis induced by H_2_O_2_ or UVB

For H_2_O_2_ treatment, the lenses of WT, Cx50KO, Cx46KO, or dKO mice were kept transparent in culture media for 24 hrs with 5% CO_2_ at 37°C before being treated with H_2_O_2_ for 4 hrs and followed by 50 μM forskolin or 2 mM 8-Br-cAMP for 20 hrs. Microscopic images were taken at identical magnification using a dissecting microscope (SM-1TSW2-L6W-5M, AmScope) after H_2_O_2_ treatment for different time points.

For UVB treatment, the lenses were kept transparent in culture media for 24 hrs with 5% CO_2_ at 37°C, and then transferred to a plate with a small shallow indentation (0.2 mm depth and 2 mm diameter) that held the lenses at a fixed distance between lenses and the UVB lamp. The lenses were exposed to 10J/cm^2^ UVB for different time periods in an incubator supplied with 5% CO_2_ at 37°C. The lenses were then treated with 50 μM forskolin, 0.1% DMSO (Ctrl), 2 mM 8-Br-cAMP, or H_2_O (Ctrl) for 4 hrs after UVB irradiation and microscopic images were captured 24 hrs after the treatment.

#### Determination of PKA activity

PKA activity was determined in the lenses of 4 months old WT, Cx50KO, Cx46KO, or dKO mice. The lenses were treated with or without 50 μM forskolin or 2 mM 8-Br-cAMP in the absence or presence of H_2_O_2_ or UVB. Briefly, lenses were homogenized with lysis buffer (0.1% protease inhibitor cocktail, 1 mM PMSF, 10 mM Na_3_VO_4_) for 30 min on ice with occasional gentle vertexing and then centrifuged at 9200 g for 10 min at 4°C. The pellet was resuspended in 1X Kinase Reaction Buffer from the PKA Colorimetric Activity Kit (EIAPKA, Invitrogen, Frederick, USA) and followed by incubation with PKA standards from the Kit or resuspended pellet samples, and reconstituted ATP in sequence. The sample-containing plates were sealed and incubated for 90 min at 30°C with shaking, and then washed three times with wash buffer from the Kit at room temperature (RT). The goat anti-rabbit IgG HRP conjugated antibody and the phosphor-PKA substrate antibody from the Kit were then added in sequence, and the plates were incubated for 60 min at RT with shaking. The plates were rinsed three times with wash buffer from the Kit at RT, and incubated with the tetramethylbenzidine substrate from the Kit, for 30 min at RT. The stop solution from the Kit was added, and the absorbance at 450 nm was measured with a microtiter plate reader (Synergy HT, Biotek, Winooski, USA).

#### Fluid flow shear stress

Fluid flow shear stress (FFSS) was used to apply mechanical stimulation on cells based on the published protocols.^30^^,^^68^^,^^69^^,^^70^ Recording medium (RM) (154 mM NaCl, 5.4 mM KCl, 1.8 mM CaCl_2_, 1 mM MgCl_2_, 5 mM D-glucose, 10 mM HEPES) was prepared as the circulating fluid. Briefly, laminar shear stress was achieved by a parallel flow chamber and a gasket with defined thickness. Gravity-driven fluid flow was generated by a peristaltic pump. The level of shear stress (1 dyne/cm^2^) was determined by the flow rate (14 mL/min) and the gasket (0.1 cm thickness and 1.4 cm width). The circulating system was pre-cleaned with double distilled water and then filled with pre-warmed RM (37°C). Cells cultured in 40-mm-diameter coverslips (Bioptechs, Butler, PA, USA) were subjected to continuous fluid flow at 1 dyne/cm^2^ for 30 min.

#### Determination of cells under apoptosis and necrosis

Cell apoptosis and necrosis were determined based on the protocol as we previously described.^25^^,^^31^ Differentiated lens primary cells were first treated with or without PKA activator, forskolin (1 μM) for 2 hrs, subjected to FFSS at 1 dyne/cm^2^ for 30 min, then incubated with 1 mM GSH for 30 min, 3 washes with RM at RT, and finally treated with 50 μM H_2_O_2_ for 4 hrs. Cell apoptosis and necrosis were detected 4 hrs post-H_2_O_2_ treatment by using FITC–Annexin V Apoptosis Detection Kit, as instructed by the manufacturer (Biolegend, San Diego, USA). The ratio of positive staining area to the total area of lentoids was used to indicate the extent of cells under apoptosis or necrosis.

#### Scrape-loading dye transfer assay

CEF cells were infected with high-titer RCAS(A) retroviral vehicle (V) or recombinant RCAS(A) retroviruses containing Cx46 or co-infected with RCAS(A) containing Cx46 and Cx50. The infected cells were grown to confluence to maximize cell–cell contact. The scrape-loading dye transfer assay was performed based on a modified procedure.^71^ Briefly, cells were scratched in the presence of two fluorescent dyes, Lucifer yellow (LY; 457 Da), which can pass through GJs channels, and rhodamine-dextran (RD; 10 kDa), which is too large to pass through GJs channels. Therefore, the presence of LY indicates cells participating in GJs coupling, and RD serves as a tracer dye for cells originally receiving the dyes. Cells were washed three times with HBSS plus 1% bovine serum albumin (BSA) for 5 min each, and then a mix containing 1% LY and 1% RD in PBS was applied, after which plates were scraped lightly with a 26.5-gauge needle. After a 15 min incubation, cells were washed with HBSS three times, twice with PBS, and then fixed in fresh 2% PFA for 30 min. Dye-transfer results were recorded by a fluorescence microscope (Keyence BZ-X710, Osaka, Japan), and the acquisition settings were kept consistent for all samples. The extent of dye transfer was measured as the ratio of LY-labeled cells to that of RD-labeled cells. At least three images per condition tested were used to assess the extent of dye transfer. The CEF cell morphology makes it difficult to visualize dye transfer through individual cells, therefore, a total area of dye transfer from the scrape loading line was measured.

#### Dye uptake assay

CEF cells were cultured in collagen-coated 40 mm-diameter coverslips at low density to minimize physical contact and transfer of dye through GJs. On the second day of culturing, CEF cells were infected with high-titer retrovirus RCAS(A), vehicle (V), or recombinant RCAS(A) containing Cx46 or co-infected with RCAS(A) containing Cx46 and Cx50. Cells were treated with or without FFSS at 1 dyne/cm^2^ for 30 min, then incubated with 0.4% LY and 0.4% RD in RM for 10 min at RT, washed with ice-cold PBS three times, and fixed by 2% PFA for 10 min. For post-FFSS dye uptake assay, 0 min, 20 min, 60 min, or 240 min after FFSS, CEF cells were incubated with 0.4% LY/RD in RM and then fixed. At least three microphotographs of fluorescence fields were captured by a fluorescence microscope (Keyence BZ-X710) with FITC and rhodamine filters. The LY uptake percentage was quantified by subtracting LY/RD double-positive cells from LY positive cells.

#### Lens frozen tissue sectioning and immunofluorescence

The lenses treated with or without H_2_O_2_ or UVB from WT, Cx50KO, Cx46KO, and dKO mice were fixed in 2% PFA for 2 hrs at RT, dehydrated with various concentrations of sucrose solution sequentially: first in 10% for 1 hr, next in 20% for 1 hr, and finally in 30% at 4°C overnight, and embedded in OCT compound (Sakura, Torrance, CA, USA). Sagittal sections (14 μm) were prepared and incubated with primary antibodies overnight at 4°C, and followed by incubation with fluorescein-conjugated secondary antibody for 1 hr at RT and 4′,6-diamidino-2-phenylindole ( DAPI ) for 5 min at RT. After rinsing with PBS three times, a drop of mounting medium was added before being covered by a coverslip. The images of specimens were taken by a fluorescence microscope (Keyence BZ-X710), and the acquisition settings were kept consistent for all samples.

#### Western blot analysis

Lenses were stored in liquid nitrogen immediately after dissection to prevent protein degradation, and then homogenized in a tapered tissue grinder with ice-cold lysis buffer (5 mM Tris, 5 mM EDTA, and 5 mM EGTA, pH 8.0) plus protease inhibitors (2 mM phenylmethylsulfonylfluoride, 5 mM N-ethylmaleimide, 1 mM Na_3_VO_4_, and 0.2 mM leupeptin). Lens homogenates were boiled in 0.6% SDS and then centrifuged at 16,600 × g for 10 min. The resulting supernatants were collected and assayed for protein concentration using the BCA protein assay reagent (Pierce, Rockford, IL, USA). To examine the expression level of superoxide dismutase 1 (SOD1) and catalase (CAT), 20–30 μg of protein was loaded on 12% or 10% SDS-PAGE, respectively, and transferred to PVDF membranes. The membranes were blocked with 5% skim milk and treated with primary antibodies: anti-SOD1 (1:300 dilution, sc-11407 Santa Cruz Biotechnology, Dallas, Texas), anti-catalase (1:300 dilution, sc-50508 Santa Cruz Biotechnology), anti-β-actin antibody (1:5000 MA5-15739, Thermo Scientific) or anti-GAPDH antibodies (1:5000 dilution, HyTest, Waltham, USA), and then detected with donkey anti-rabbit IgG conjugated IRDye® 800CW, or donkey anti-mouse IgG conjugated IRDye® 680RD (LI-COR Biosciences, Lincoln, USA) (1:15000 dilution) using a Licor Odyssey Infrared Imager (Lincoln, NE, USA). The intensity of the bands was quantified using Image Studio Lite Ver 5.2 software.

#### Determination of intracellular ROS

Intracellular ROS levels in mouse lenses were studied by using carboxy H2DCFDA, a general oxidative stress indicator. The lenses of WT, Cx50KO, Cx46KO, or dKO mice were kept transparent in culture media for 24 hrs with 5% CO_2_ at 37°C before being treated with or without 0.5 mM H_2_O_2_ for 4 hrs and followed by incubation with or without 50 μM forskolin for 2 hrs. Lens were then incubated with 10 μM carboxy H2DCFDA for 30 min at 37°C and rinsed three times at RT with pre-warmed artificial aqueous humor (AAH) (125 mM NaCl, 4.5 mM KCl, 10 mM NaHCO_3_, 2 mM CaCl_2_, 0.5 mM MgCl_2_, 5 mM D-glucose, 20 mM sucrose buffered with 10 mM HEPES to pH 7.4). At least three microphotographs of fluorescence fields were captured by a fluorescence microscope (Keyence BZ-X710) with a FITC filter. The mean fluorescence intensity was quantified by using NIH Image J software.

### Quantification and statistical analysis

All data were analyzed with GraphPad Prism 7 Software (GraphPad Software, La Jolla, CA). The data were expressed as the mean ± SEM of at least three measurements. Statistical significance was designated for analyses with p < 0.05. The numbers of samples (N) are plotted explicitly in each graph. Asterisks in all figures indicate the degree of significant differences compared to controls, ∗, p < 0.05; ∗∗, p < 0.01; ∗∗∗, p < 0.001; ∗∗∗∗, p < 0.0001.

## Data Availability

• Data: all data generated or analyzed during this study are included in this article and its supplemental information files. Any additional information required to re-analyze the data reported in this article is available from the lead contact upon request. • Code: This paper does not report original code. • All other requests: Any additional information required to reanalyze the data reported in this paper is available from the lead contact upon request.
