# Supplementary material for: Protein kinase A activation alleviates cataract formation via increased gap junction intercellular communication
Source: iScience. 2023 Feb 2;26(3):106114. doi: 10.1016/j.isci.2023.106114 (PMC9958365; doi:10.1016/j.isci.2023.106114)
Supplement: Document S1. Figures S1–S5 [file mmc1.pdf]

iScience, Volume *26*

Supplemental information

Protein kinase A activation alleviates cataract formation via increased gap junction intercellular communication

Yu Du, Yuxin Tong, Yumeng Quan, Guangyan Wang, Hongyun Cheng, Sumin Gu, and Jean X. Jiang

Supplemental figure legends:

Figure S1. H_2O_2-induced cataract formation in response to different concentrations of PKA activator in WT mice, related to Figure 3 and Figure 4. The lens of WT mice were kept transparent in culture media for 24 hrs with 5% CO_2 at 37°C before being treated with 0.5 mM H_2O_2 for 4 hrs, followed by treatment with or without different concentrations of forskolin **(A)** or 8-Br-cAMP **(B)**. The opacity intensity was quantified by NIH Image J software. The data are presented as the mean ± SEM. ($n \geq 3$). *, $P < 0.05$; **, $P < 0.01$; ***, $P < 0.001$ ****, $P < 0.0001$ (Two-way ANOVA).

Figure S2. Dye transfer with different concentrations of forskolin, related to Figure 6. CEF cells were infected with high-titer RCAS(A) retroviral vehicle (V) or recombinant RCAS(A) retroviruses containing Cx46 or co-infected with RCAS(A) containing Cx46 and Cx50, and cells were grown to confluence to maximize cell-cell contact. **(A)** Cells were treated with different concentrations of forskolin for 2 hrs before scrape loading dye transfer assay using LY (green) as a probe for gap junction coupling and RD (red) for detecting originally dye-loaded cells. Scale bar: 50 μm. **(B)** The extent of dye transfer was measured as the ratio of LY-stained cells to that of RD-labeled cells. The data are presented as the mean ± SEM. (n=3). ****, $P < 0.0001$ (Two-way ANOVA).

Figure S3. Antioxidant protein expression in WT, Cx46KO, Cx50KO, and dKO mouse lens, related to Figure 7. The protein extracts of isolated lenses from WT, Cx46KO, Cx50KO, and dKO mice were immunoblotted with anti-SOD1 **(A)**, catalase **(B),** or β-actin **(A and B)** antibodies. The intensity of the bands was quantified using an Image Studio Lite Ver 5.2

software, and the ratio of SOD1 to β-actin **(A, lower panel)** and catalase to β-actin **(B, lower panel)** was presented. The data are presented as the mean ±SEM. (n = 4). *, P < 0.05; **, P < 0.01; ***, P < 0.001; ****, P < 0.0001 (One-way ANOVA).

Figure S4. Anti-oxidative protein in different regions of lenses, related to Figure 7. (A) The lenses from WT, Cx46KO, Cx50KO, and dKO mice were treated with 300 μM H_2O_2 or 10 J/cm^2 UVB, and cryo-lens sections were immunostained with anti-SOD1 (green, upper panel) or anti-catalase (red, lower panel) antibody. High resolution fluorescence images of anterior (AT), equator (EQ), posterior (PT), and nuclear (NC) regions of lenses were capatured. Scale bar = 20 μm. The lens regions are indicated by dotted frames on the schematic diagram. **(B)** The intensity of the SOD1 or catalase was quantified by NIH Image J software. The data are presented as the mean ±SEM. (n = 3). *, P < 0.05; **, P < 0.01; ***, P < 0.001; ****, P < 0.0001 (One-way ANOVA).

Figure S5. Forskolin protects lens and reduces ROS against H_2O_2 in a time-dependent manner, related to Figure 8. (A) The lens of WT mice was kept transparent in culture media for 24 h at 37°C before being treated with 0.5 mM H_2O_2 for 4 hrs, followed by treatment with 50 μM forskolin or vehicle (DMSO). Images were taken at the identical magnification 5, 6, 8, 12, or 24 hrs after H_2O_2 treatment using a dissecting microscope to count the increase in lens opacity. Lens were then incubated with 10 μM carboxy H2DCFDA for 30 min at 37°C. At least three microphotographs of fluorescence fields were captured by a fluorescence microscope (Keyence BZ-X710) with a FITC filter. Scale bar: 100 μm. The mean fluorescence intensity was quantified by using NIH Image J. **(B)** Intracellular ROS level and **(C)** the lens opacity was quantified by

NIH Image J software. The data are presented as the mean ± SEM. (n=3). *$P < 0.05$, **$P < 0.01$, ***$P < 0.001$, ****$P < 0.0001$ (Two-way ANOVA).

Fig. S1

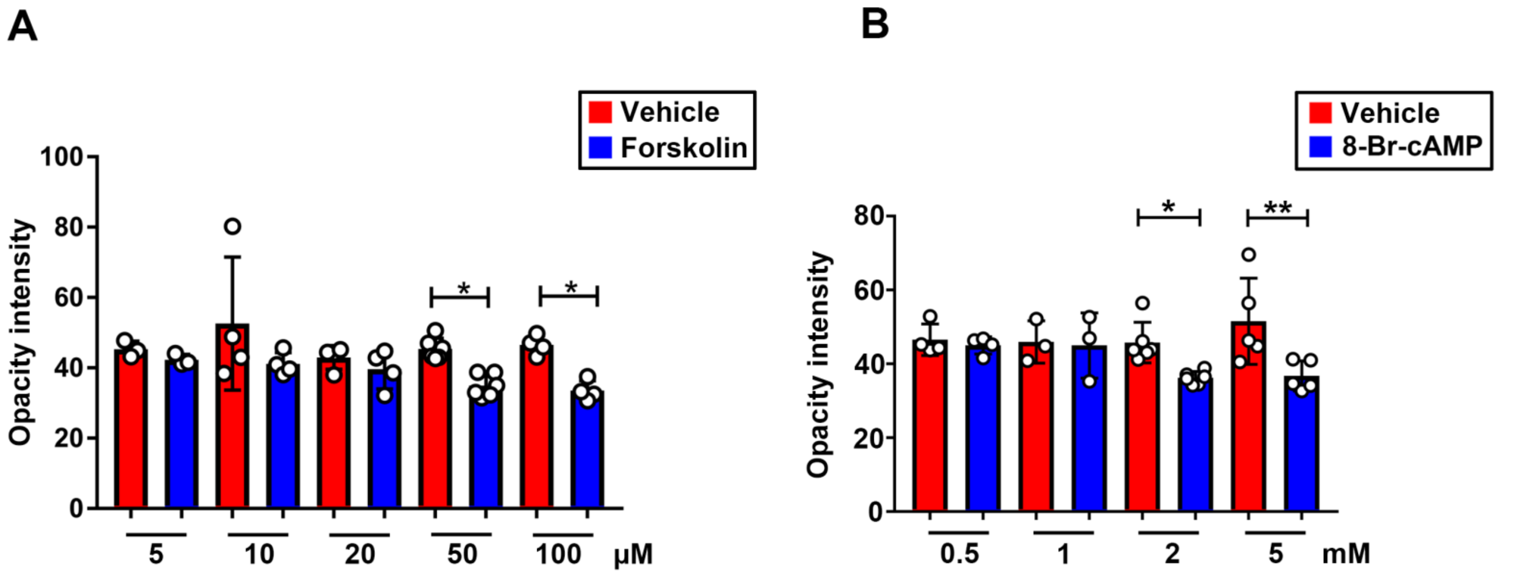

A
Vehicle
Forskolin
Opacity intensity
100
80
60
40
20
0
*
*
5
10
20
50
100
μM
B
Vehicle
8-Br-cAMP
Opacity intensity
80
60
40
20
0
*
**
0.5
1
2
5
mM

Fig. S2

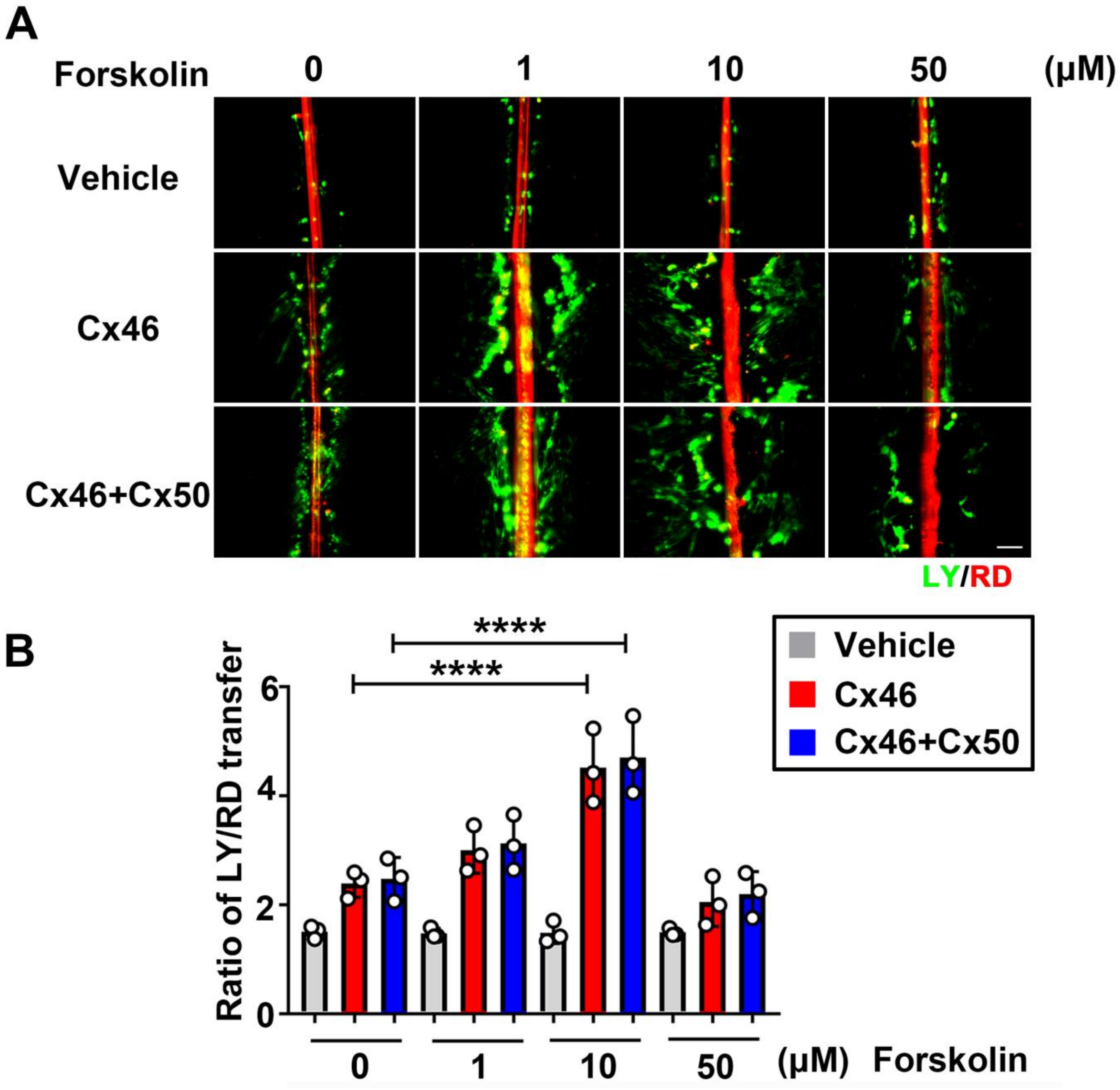
A
Forskolin
0
1
10
50
(μM)
Vehicle
Cx46
Cx46+Cx50
LY/RD
B
Ratio of LY/RD transfer
****
****
Vehicle
Cx46
Cx46+Cx50
6
4
2
0
0
1
10
50
(μM) Forskolin

Fig. S3

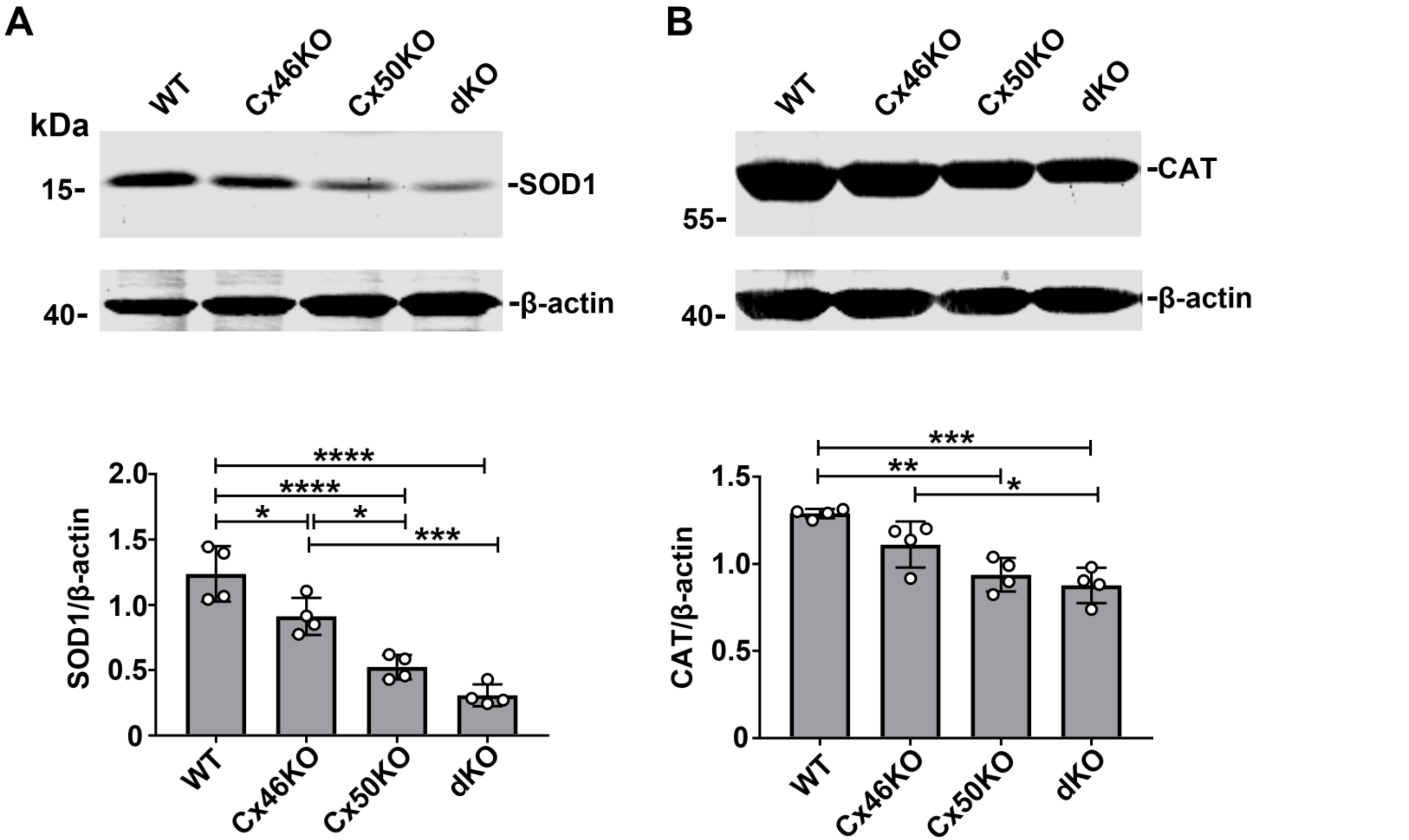
A
WT
Cx46KO
Cx50KO
dKO
kDa
15-
-SOD1
40-
-β-actin
B
55-
-CAT
40-
-β-actin
SOD1/β-actin
2.0
1.5
1.0
0.5
0
****
****
*
*
***
CAT/β-actin
1.5
1.0
0.5
0
***
**
*

Fig. S4

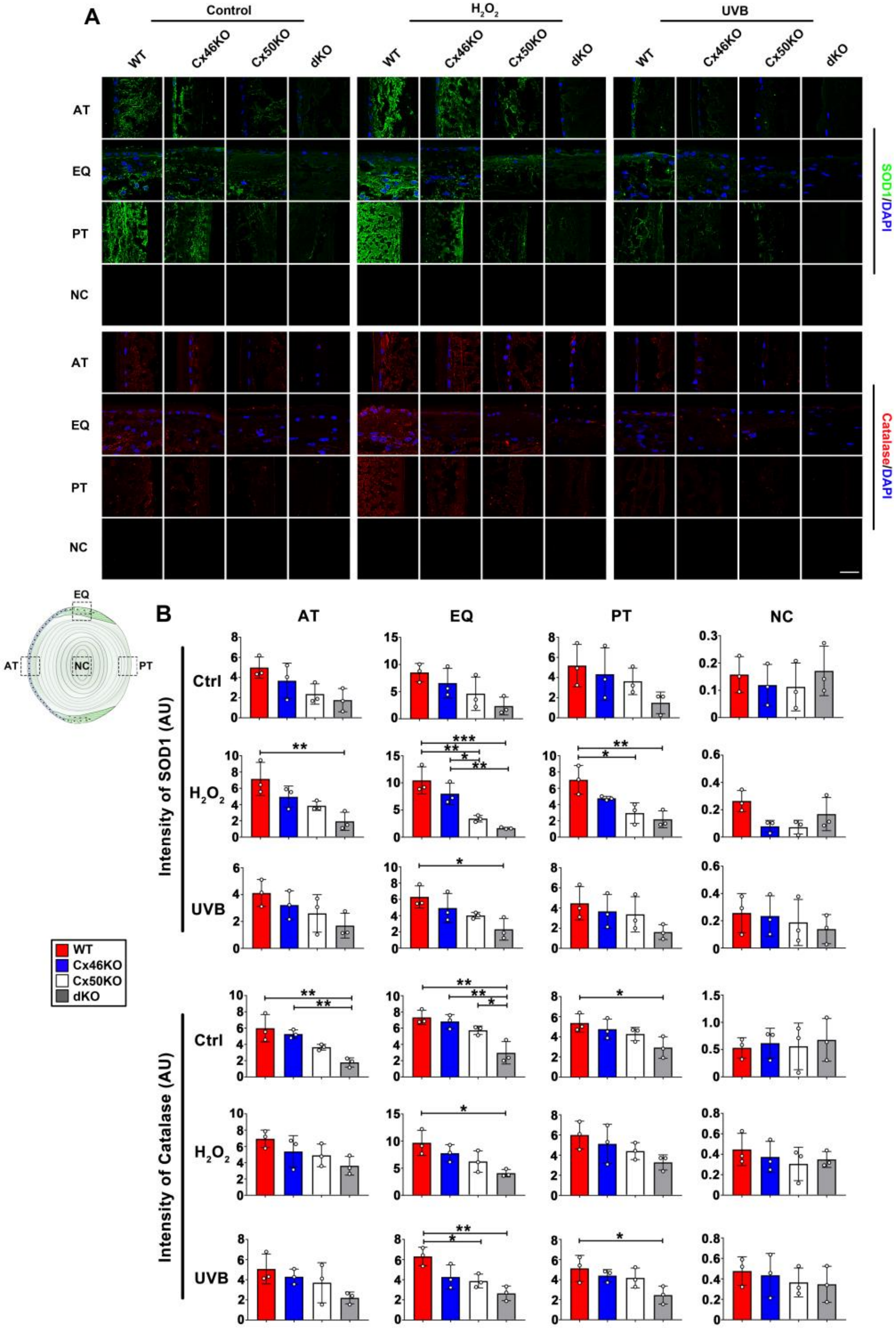

A
Control
H_2O_2
UVB
WT
Cx46KO
Cx50KO
dKO
AT
EQ
PT
NC
SOD1/DAPI
Catalase/DAPI
B
AT
EQ
PT
NC
Ctrl
H_2O_2
UVB
Intensity of SOD1 (AU)
Intensity of Catalase (AU)
WT
Cx46KO
Cx50KO
dKO

Fig. S5

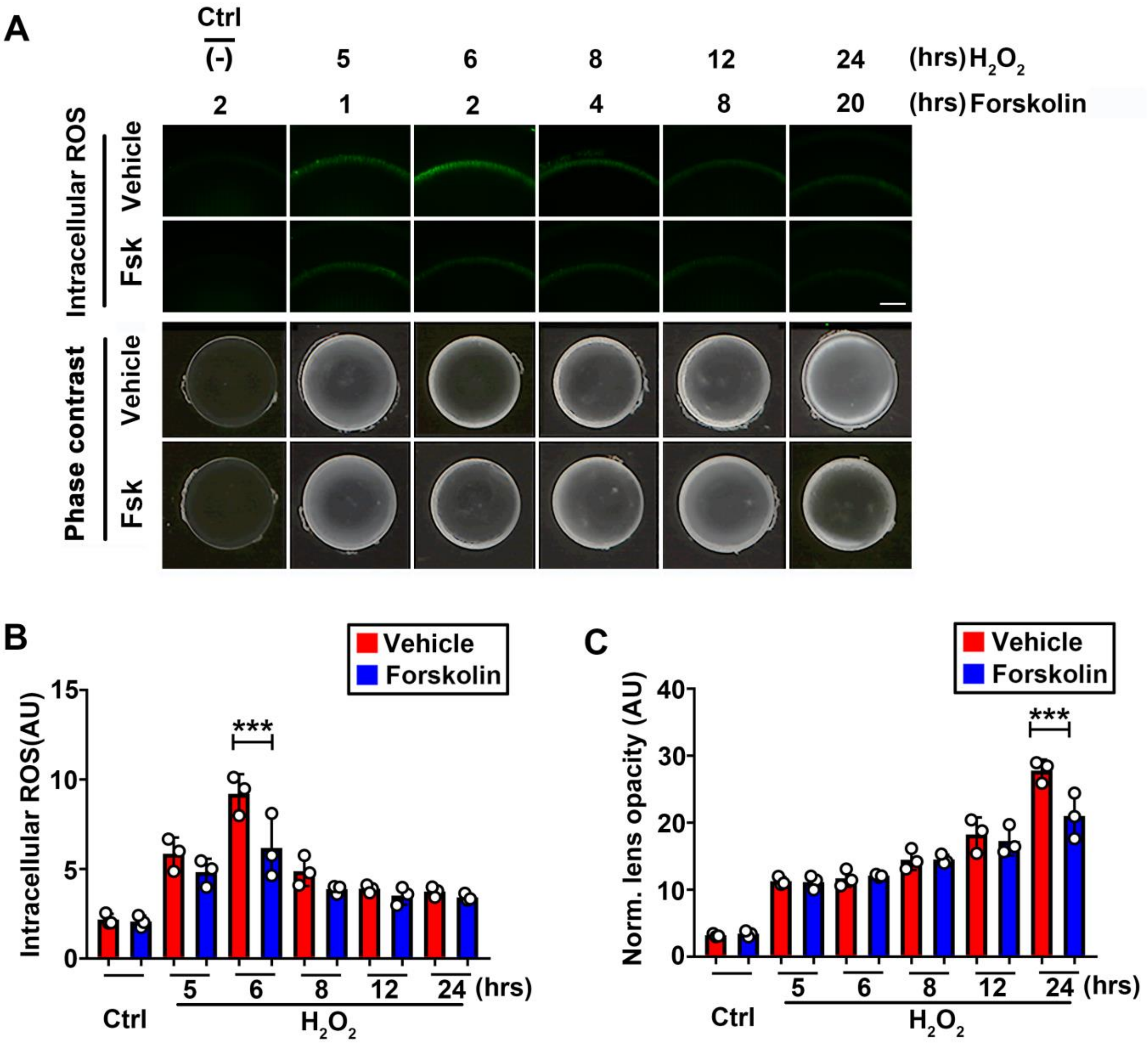
A
Ctrl
(-)
5
6
8
12
24
(hrs) H_2O_2
2
1
2
4
8
20
(hrs) Forskolin
Intracellular ROS
Vehicle
Fsk
Phase contrast
Vehicle
Fsk
B
Vehicle
Forskolin
Intracellular ROS(AU)
15
10
5
0
***
Ctrl
5
6
8
12
24 (hrs)
H_2O_2
C
Vehicle
Forskolin
Norm. lens opacity (AU)
40
30
20
10
0
***
Ctrl
5
6
8
12
24 (hrs)
H_2O_2
